# *Mycobacterium tuberculosis* Induces an Atypical Cell Death Mode to Escape from Infected Macrophages

**DOI:** 10.1371/journal.pone.0018367

**Published:** 2011-03-31

**Authors:** Jinhee Lee, Teresa Repasy, Kadamba Papavinasasundaram, Christopher Sassetti, Hardy Kornfeld

**Affiliations:** 1 Department of Medicine, University of Massachusetts Medical School, Worcester, Massachusetts, United States of America; 2 Department of Microbiology and Physiological Systems, University of Massachusetts Medical School, Worcester, Massachusetts, United States of America; 3 Howard Hughes Medical Institute, University of Massachusetts Medical School, Worcester, Massachusetts, United States of America; Fundació Institut Germans Trias i Pujol; Universitat Autònoma de Barcelona CibeRES, Spain

## Abstract

**Background:**

Macrophage cell death following infection with *Mycobacterium tuberculosis* plays a central role in tuberculosis disease pathogenesis. Certain attenuated strains induce extrinsic apoptosis of infected macrophages but virulent strains of *M. tuberculosis* suppress this host response. We previously reported that virulent *M. tuberculosis* induces cell death when bacillary load exceeds ∼20 per macrophage but the precise nature of this demise has not been defined.

**Methodology/Principal Findings:**

We analyzed the characteristics of cell death in primary murine macrophages challenged with virulent or attenuated *M. tuberculosis* complex strains. We report that high intracellular bacillary burden causes rapid and primarily necrotic death via lysosomal permeabilization, releasing hydrolases that promote Bax/Bak-independent mitochondrial damage and necrosis. Cell death was independent of cathepsins B or L and notable for ultrastructural evidence of damage to lipid bilayers throughout host cells with depletion of several host phospholipid species. These events require viable bacteria that can respond to intracellular cues via the PhoPR sensor kinase system but are independent of the ESX1 system.

**Conclusions/Significance:**

Cell death caused by virulent *M. tuberculosis* is distinct from classical apoptosis, pyroptosis or pyronecrosis. Mycobacterial genes essential for cytotoxicity are regulated by the PhoPR two-component system. This atypical death mode provides a mechanism for viable bacilli to exit host macrophages for spreading infection and the eventual transition to extracellular persistence that characterizes advanced pulmonary tuberculosis.

## Introduction

Following inhalation by a naïve host, *Mycobacterium tuberculosis* (Mtb) enters lung macrophages, which provide an intracellular environment necessary to support bacterial growth. To preserve this replication sanctuary virulent Mtb strains inhibit extrinsic, tumor necrosis factor (TNF)-α mediated apoptosis (a potential host defense against intracellular pathogens) through functions of the mycobacterial *nuoG* [1] and *secA2* [2] genes and superoxide dismutase A [3]. The capacity of Mtb to suppress apoptosis implies the existence of a mechanism for bacilli to escape from macrophages whose utility is expended. Park et al. [4] reported that infection of murine bone marrow-derived macrophages at low multiplicity of infection (MOI 5) resulted in cell death 6 days later, at which point the intracellular bacillary load was ∼18 per macrophage. Mtb strains with intrinsically slow intracellular growth rates were not cytotoxic in this time frame. The survival of macrophages challenged with potentially cytotoxic strains was preserved by pretreatment with interferon (IFN)-γ that suppressed bacterial replication. Their data showed that a low intracellular burden of virulent Mtb does not promote macrophage cell death, at least within 6 days, and suggested that cytotoxicity occurs when Mtb replication exceeds a threshold intracellular bacillary load.

The concept that macrophage cell death depends at least in part on intracellular bacillary load was supported by MOI dose-response studies demonstrating rapid cytotoxicity induced by virulent Mtb Erdman when the intracellular bacillary load exceeded a threshold of ∼20 per macrophage, corresponding to MOI 25 [5]. In contrast *M. smegmatis* was minimally cytotoxic even at MOI 50. Different from classical apoptosis, macrophage cell death induced by Erdman was independent of TNF-α and caspases. Dying macrophages showed apoptotic features of nuclear condensation and phosphatidylserine (PS) translocation to the outer cell membrane leaflet within 3 h of infection, but progressed rapidly to necrosis identified by propidium iodide (PI) staining. This form of infection-induced cell death was consistent with a mycobacterial exit mechanism but its features, causal mechanism and relation to other instances of infection-induced cell death were not defined.

In the present study we investigated the characteristics and determinants of macrophage cell death caused by virulent Mtb at high MOI. We show that death is preceded by lysosomal membrane permeabilization (LMP) followed by widespread destruction of lipid bilayers and concomitant degradation of several phospholipid species with at least partial involvement of lysosomal lipases. Unlike many other examples of lysosomal cell death, that caused by Mtb does not depend on cathepsins B, L or D. Disruption of outer and inner mitochondrial membranes occurs in the absence of pro-apoptotic Bax or Bak, and is followed by collapse of the mitochondrial transmembrane potential and depletion of cellular ATP. Mtb-induced cell death occurs in the absence of caspase-1 or activated cathepsin B and is therefore different from pyroptosis or pyronecrosis that are death modes induced by certain other intracellular bacterial pathogens. The cytotoxicity of Mtb did not depend on the reported membrane-disruptive function encoded by genes of the mycobacterial RD1 region [6]. Instead, we found that inactivating the PhoPR two-component system of Mtb profoundly reduced the induction of LMP, mitochondrial injury and cell death at high MOI. Our study reveals a novel cell death mechanism mediated by lysosomal lipases and defines a role for one or more PhoPR-regulated genes in TB pathogenesis beyond the previously described inhibition of phagolysosome fusion and secretion of early secretory antigenic target-6 (ESAT-6) [7].

## Results

### Ultrastructural features of Mtb-induced macrophage cell death

We previously reported that virulent Mtb at MO I 25 (corresponding to an intracellular load of ∼20 bacilli per macrophage) rapidly induces cell death in a caspase-independent manner [5]. This mode of death occurs many days after low MOI challenge when bacillary replication pushes the intracellular load past a toxic threshold [4], but is more conveniently studied by presenting macrophages with a MOI (≥25) sufficient to reach the threshold value immediately. The high MOI approach permits partial synchronization of cell death in the cultured cells and also bypasses the limitation of certain mycobacterial strains (e.g. *M. bovis* BCG and *phoPR* mutants) to multiply within macrophages following low MOI challenge [7]. The relevance of high burden infection to TB disease in vivo was demonstrated by acid fast staining of lung leukocytes harvested from mice 2 wk after aerosol infection with 100 CFU Erdman delivered to the lung (Fig. S2). Approximately 3% of alveolar macrophages from Mtb-infected mice contained >20 bacilli, and ∼15% contained 16–20 bacilli at that time point.

**Figure 1:**
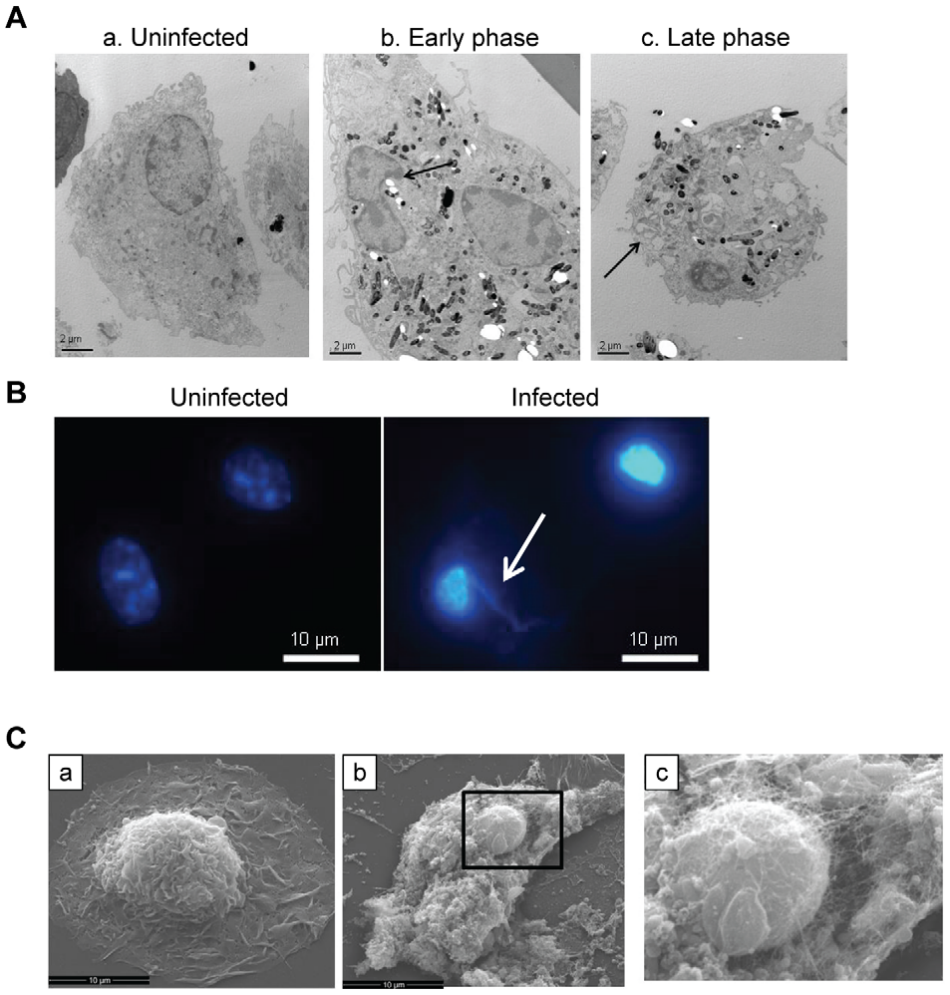
Mtb induces a morphologically unique form of macrophage cell death. (A) TEM images of uninfected macrophages (a) and macrophages infected with Mtb (MOI 25) for 3 h (b and c). Typical early changes (b) were peripheral nuclear condensation and partial nuclear protrusion from condensed areas (*arrow* [b]) while cells at the late phase of death had ruptured plasma membranes (*arrow* [c]) and advanced nuclear pyknosis. Micrographs are representative of three independent experiments. Bar  = 500 nm. (B) DAPI staining shows nuclear pyknosis and extrusion of chromosomal DNA (*arrow*) in a macrophage infected with Mtb. Bar  = 10 µm. (C) SEM images of uninfected (a) and Mtb-infected macrophages (b,c) showing extensive plasma membrane loss in the latter. The boxed area in b is shown with 3X zoom in c. Bar  = 10 µm.

To further define the characteristics of cell death induced by virulent Mtb we examined infected macrophages by transmission and scanning electron microscopy (TEM and SEM). Morphological changes were distinct from typical apoptosis or necrosis but showed some features of both death modes. The earliest changes, widely present by 3 h post-infection, included peripheral nuclear chromatin condensation and protrusion of nuclear membranes from areas of chromatin condensation (Fig. 1A); changes associated with apoptosis [13]. Unlike typical apoptosis, chromatin condensation did not progress to more complete crescentic or globular forms and nuclear fragmentation was not observed. Instead, condensed nuclei shrank and in some cells disappeared with progression of the death process (Fig. 1A). Apoptotic vesicle formation was absent in these dying cells. A more advanced phase of death was observed in some cells as early as 3 h post-infection and in most cells by 18 h. These cells showed plasma membrane disruptions that define necrosis, but without increased cell volume typical of osmotic lysis. This suggested that membrane disruption was a primary event rather than a consequence of swelling after failure of membrane ion pumps. TEM revealed widespread membrane damage as a characteristic feature of this death mode. We observed disruption of phagosomal membranes with intrusion of cytosolic material into the phagosome lumen (Fig. S3A) and in some cases bacilli were present in the cytosol without a visible phagosomal membrane boundary, as others reported [14]. Disruption of nuclear envelopes was also observed; the nuclear envelope of some Mtb-infected cells had indistinct boundaries (Fig. S3A) with leakage of chromosomal DNA into the cytosol (Fig. 1B). Plasma membrane damage seen by TEM was also evident on SEM imaging. As early as 3 h after infection some macrophages had extensive loss of plasma membranes, exposing the underlying cytoskeleton and intracellular organelles (Fig. 1C). The proportion of cells with this appearance increased over time, and bacilli could be seen spilling out of cells (Fig. S3B).

### Bax/Bak independent damage of mitochondrial outer and inner membranes

**Figure 2:**
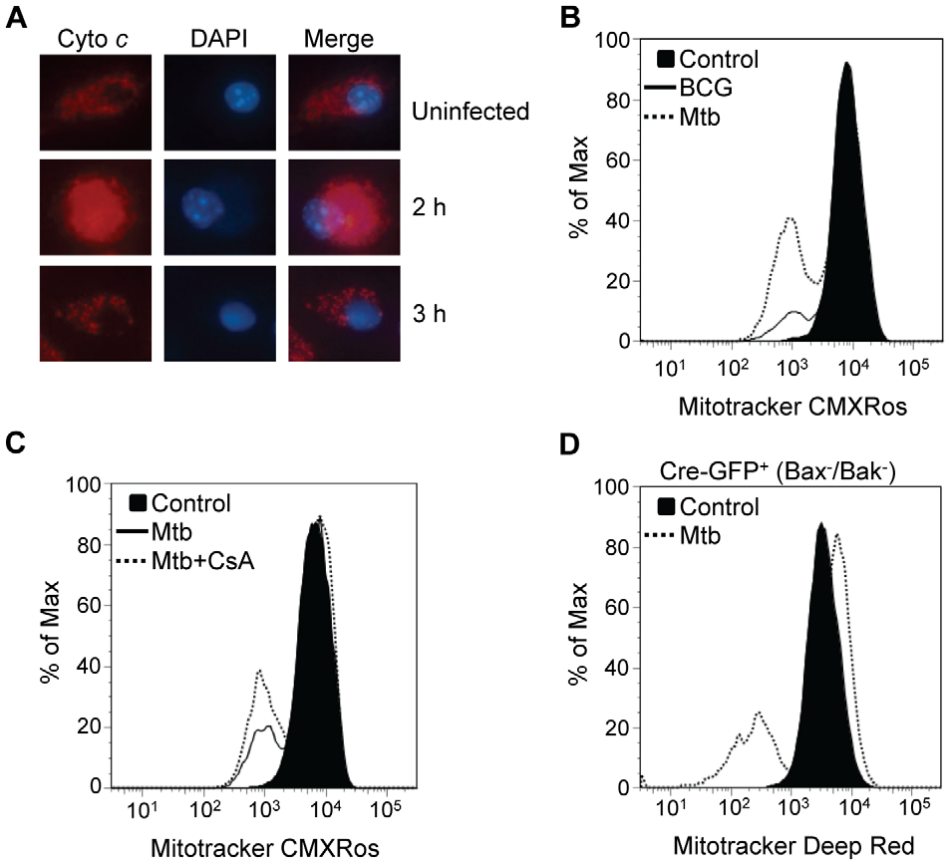
Bax/Bak-independent mitochondrial injury in Mtb-infected macrophages. (A) Macrophages infected with *M. bovis* BCG (MOI 25, 2 h and 3 h) were stained with anti-cytochrome *c* mAb and DAPI for fluorescence microscopy (X400). Micrographs are representative of two independent experiments. (B) Loss of ΔΨ_m_ in macrophages infected for 3 h with BCG or Mtb measured by flow cytometry with Mitotracker CMXRos. Histograms are representative of three independent experiments. (C) Macrophages were infected with Mtb (3 h) in the presence of absence of CsA (5 µM) and probed with Mitotracker dye CMXRos to measure ΔΨ_m_. Histograms are representative of three independent experiments. (D) Macrophages lacking Bax and Bak were infected with Mtb and probed with Mitotracker Deep Red to measure ΔΨ_m_. Histograms are representative of three independent experiments.

Mitochondria play a central role in determining cell fate [15]. We investigated mitochondrial integrity in macrophages at time points after Mtb infection by staining with anti-cytochrome *c* antibody. Cells were counterstained with DAPI to assess nuclear morphology. Flow cytometric measurement of cationic dye retention was used to assess mitochondrial transmembrane potential. Cytochrome *c* was confined within normal mitochondria of uninfected cells that also had normal appearing nuclei (Fig. 2A). Cytosolic translocation of cytochrome *c* indicating mitochondrial outer membrane permeabilization (MOMP) was first evident 2 h after infection, while nuclear morphology was unchanged at that time. By 3 h post-infection residual cytochrome *c* staining revealed distended mitochondria, reflecting terminal dissipation of the transmembrane potential (ΔΨ_m_). Nuclear condensation was evident at this stage. The sequence of events indicates that MOMP precedes the loss of ΔΨ_m_ and the earliest appearance of apoptotic nuclear morphology. Dissipation of ΔΨ_m_ 3 h after infection was confirmed by flow cytometry (Fig. 2B) and fluorescence microscopy (Fig. S4A) using the cationic dye Mitotracker CMXRos. It was accompanied by depletion of intracellular ATP (Fig. S4B). Heat-killed Mtb failed to disrupt ΔΨ_m_ (Fig. S4C) which was consistent with our previous observation that only viable bacilli kill macrophages [5].

In different contexts of cell death, collapse of ΔΨ_m_ may develop immediately after MOMP (sometimes in a caspase-dependent manner) or alternatively at a later point during the execution phase of apoptosis. A major mechanism for loss of ΔΨ_m_ is formation of the mitochondrial permeability transition (PT) pore, a protein complex whose main components are voltage dependent anion channel, adenine nucleotide translocase, and cyclophilin-D, and whose opening can be regulated by Bax. The cyclophilin-D inhibitor cyclosporine A (CsA) blocks Bax-dependent PT opening [16] and promotes cell survival [17]. We confirmed that CsA blocks chemically-induced mitochondrial PT complex formation (Fig. S5) and then tested whether it could prevent dissipation of ΔΨ_m_ in macrophages heavily infected with Mtb. As shown in Fig. 2C, CsA failed to suppress cationic dye release, indicating that PT complex formation is not responsible for ΔΨ_m_ dissipation in this setting. The inability of CsA to prevent cationic dye release is consistent with our previous finding that CsA does not prevent Mtb-induced cytolysis [5].

The pro-apoptotic Bcl-2 family members Bax and Bak are essential factors in the regulated MOMP that characterizes the intrinsic apoptosis pathway [18]. We next examined whether Bax and Bak are required for MOMP induced by Mtb. Macrophages from mice with targeted mutation of *bak* plus a floxed *bax* locus were transduced with a retrovirus expressing Cre recombinase. The resulting Bax/Bak deficient macrophages resisted staurosporine-induced ΔΨm dissipation as expected (data not shown) but were fully susceptible to Mtb-induced ΔΨm dissipation (Fig. 2D). These results indicate that mitochondrial injury results from host and/or pathogen factors acting directly on outer and inner mitochondrial membranes without the involvement of Bcl-2 family or PT pore complex proteins. Candidates for this type of effect include microbial factors such as the *Helicobacter pylori* vacA toxin [19] or host factors such as lysosomal hydrolases [20], [21]. Finding no evidence of Mtb gene products containing a mitochondrial targeting sequence similar to that of the *H. pylori* VacA toxin p34, we directed our attention to lysosomal hydrolases and LMP.

### Mtb induces LMP

**Figure 3:**
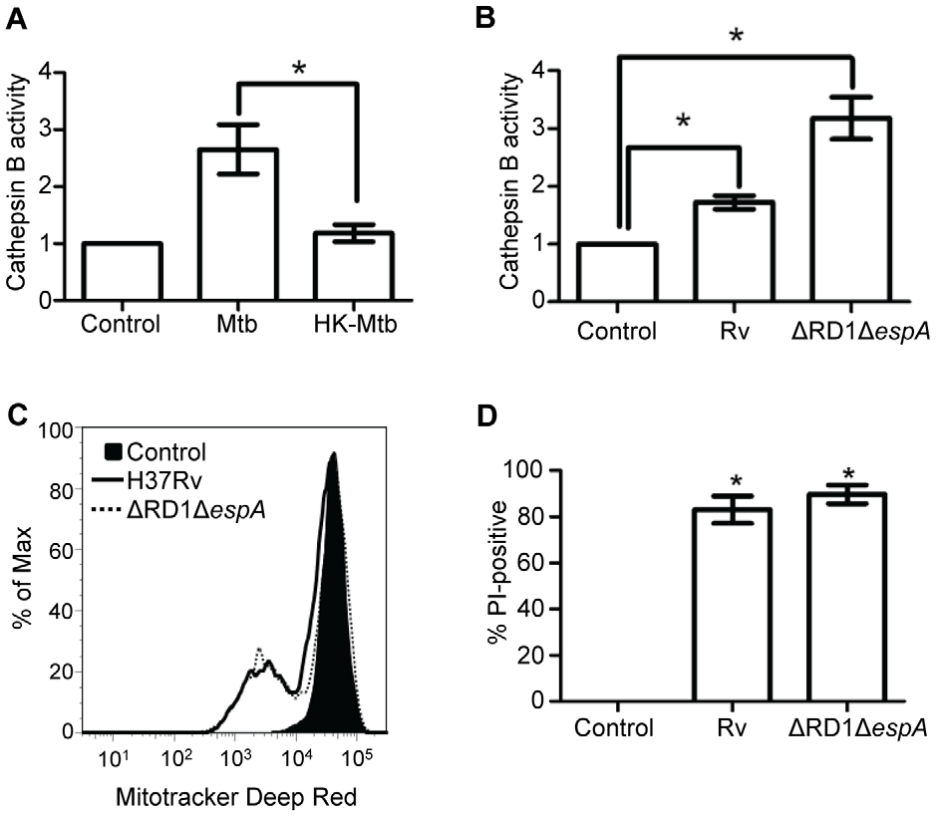
Mtb infection induces LMP independent of the ESX1 system. (A) Cytosolic cathepsin B activity in macrophages were challenged with viable or heat-killed (*HK*) Mtb for 2 h and then cytosolic extracts were prepared for measurement of cathepsin B activity indicative of LMP. Results are expressed as the fold increase of cathepsin B activity relative to uninfected (*control*) cells, normalized by the fold increase in LDH compared to uninfected cells (*P<0.05; error bars, ± SD). (B) Cytosolic cathepsin B activity in macrophages challenged with RvΔRD1Δ*espA* or the parental strain H37Rv (*P<0.05). (C) Macrophages were challenged with RvΔRD1Δ*espA* or H37Rv (3 h) and probed with Mitotracker DeepRed developed mitochondrial injury (ΔΨ_m_ dissipation) comparable to parental H37Rv. Histograms are representative of three independent experiments. (D) RvΔRD1Δ*espA* induces macrophage cell death (PI positivity) comparable to H37Rv (18 h; *P<0.05 compared to uninfected cells).

Loss of lysosomal limiting membrane barrier function permits cytosolic translocation of potentially damaging hydrolases normally sequestered in the lysosome. Others have shown that Mtb can induce LMP at least under certain conditions [14], [22]. To confirm that Mtb causes LMP under the conditions of our experiments we measured cytosolic translocation of cathepsin B activity. Infection with Mtb increased cytosolic cathepsin B activity compared to uninfected cells in a dose-dependent manner (Fig. S6A). Cytosolic cathepsin B activity was not increased by challenge with heat-killed Mtb (Fig. 3A), thus linking LMP with cell death that also requires viable intracellular bacilli for its induction [5].

The ESX1 secretion system encoded by genes in the RD1 locus of Mtb has been implicated in membrane damage by the pore-forming function of ESAT-6 [6] and in the escape of mycobacteria from phagosomes [14], making the ESX1 system a plausible candidate for LMP and cytotoxicity in our experiments. ESAT-6 secretion depends on ESX1 as well as the function of a secreted protein encoded by the chromosomally distant *espA* gene [9]. Unexpectedly, we found that cytosolic translocation of cathepsin B was induced by a mutant lacking both RD1 and *espA* (RvΔRD1Δ*espA*; Fig. 3B). This indicated that the ESX1 system is not required for LMP in the setting of high intracellular bacterial burden, a conclusion supported by finding no difference in LMP induction between BCG (which lacks the RD1 locus) or Erdman (Fig. S6B). Infection with RvΔRD1Δ*espA* also caused cationic dye release (Fig. 3C) and cell death (Fig. 3D) at levels comparable to the parental strain. While previous MOI dosing experiments indicated that BCG was somewhat less cytopathic than Edrman [5], the present experiments conclusively demonstrated that LMP, mitochondrial injury and macrophage cytolysis do not require the ESX1 system.

### Cathepsins and caspase-1 are not required for Mtb-induced cell death

**Figure 4:**
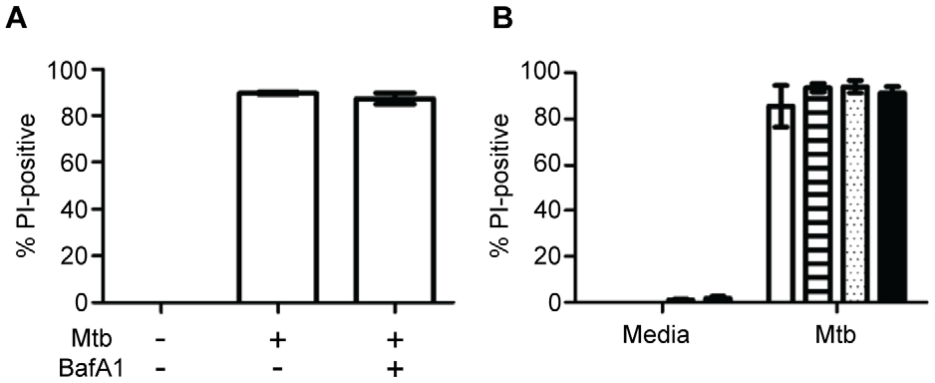
Mtb cytotoxicity does not depend on cathepsins B or L. (A) Macrophages pretreated with BafA1 or buffer control where challenged with Mtb (18 h) and then cell death was quantified by PI staining and microscopy (*P<0.05). (B) Cell death 18 h after Mtb challenge was measured by PI staining in wildtype macrophages treated with control siRNA (*empty bar*), wildtype macrophages treated with cathepsin B siRNA (*striped bar*), cathepsin L null macrophages treated with control siRNA (*speckled bar*) and cathepsin L null macrophages treated with cathepsin B siRNA (*filled bar*).

Cell death is a recognized consequence of LMP that has most often been attributed to release of activated proteases into the cytosol, particularly cathepsins B, D and L [23]. Cathepsin B is linked to several lysosomal cell death pathways including pyronecrosis in *Shigella* infection [24]. We previously reported that inhibitors of cathepsins B and L, but not cathepsin D, partially rescued macrophages from Mtb-induced death [5]. Interpretation of those experiments was limited by the questionable specificity of these agents and their toxicity at the concentrations needed to observe any protective effect. As an alternative approach we used the vesicular proton pump ATPase inhibitor bafilomycin A1 (BafA1) to inhibit lysosome acidification (Fig S7A), which is required to enable the proteolytic activity of cathepsins (Fig. S7B) and to prevent cathepsin-mediated cell death following treatment with the lysosomotropic agent chloroquine (S7C). Pretreating macrophages with BafA1 failed to prevent Mtb cytotoxicity (Fig. 4A). Furthermore, we found no protection after silencing cathepsin B in wildtype or in cathepsin L null macrophages (Fig. 4B). These results do not exclude the involvement of the many other lysosomal hydrolases in the cytotoxicity of Mtb but they establish that this death mode is distinct from pyronecrosis and does not depend on lysosomal proteases typically linked to cell death.

**Figure 5:**
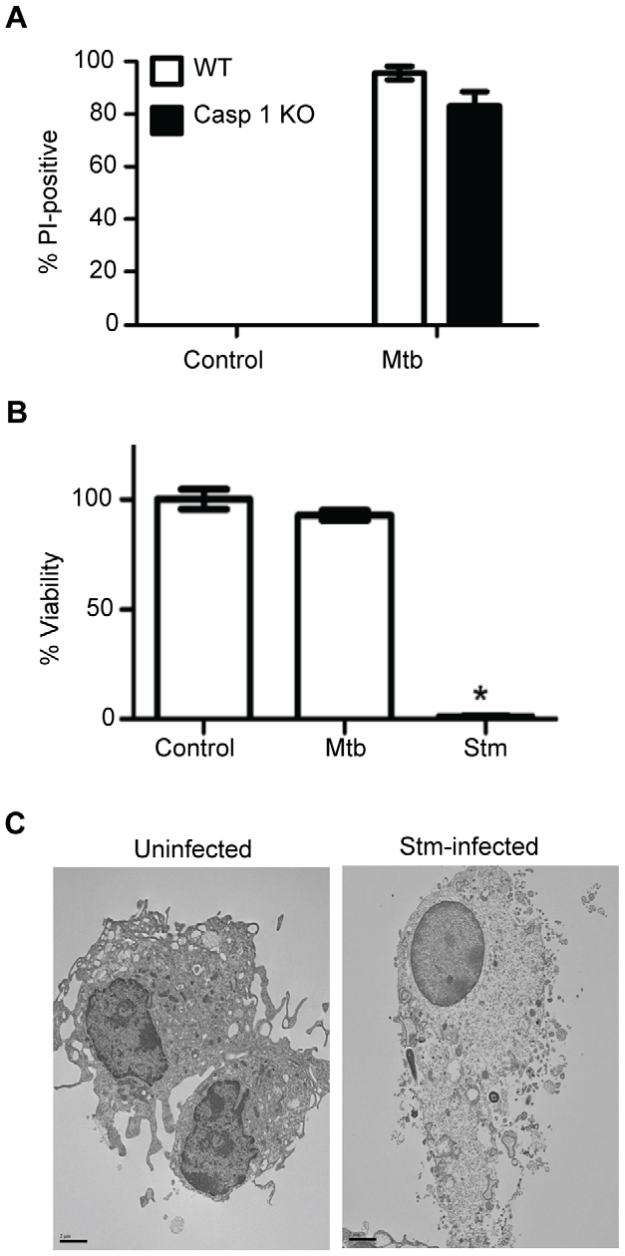
Mtb does not kill macrophages by pyroptosis. (A) Caspase-1 null (*Casp 1 KO*) and wildtype (*WT*) macrophages on the same NOD genetic background were infected with Mtb (18 h). Cell death was measured by PI incorporation (*P<0.05). (B) C57BL/6 macrophages were infected with *S. typhimurium* (Stm) or Mtb, both at MOI 25 for 4 h. Cell death was measured by neutral red dye uptake (*P<0.05 compared with uninfected or Mtb-infected macrophages. (C) TEM image of *S. typhimurium*-infected macrophage (MOI 25, 2 h) showing evidence of osmotic lysis with preserved nuclear morphology. Bar  = 2 µm.

Pyroptosis is distinct death mode that may be induced by *Shigella*, *Salmonella*, *Francisella* or *Legionella* infection in a strictly caspase-1-dependent manner [25], [26]. Macrophages from caspase-1 null mice were equally susceptible as wild type macrophages to Mtb-induced cell death (Fig. 5A), excluding pyronecrosis as the causal mechanism. This conclusion was further supported by evidence that *Salmonella* cytotoxicity operates with much faster kinetics than Mtb (Fig. 5B) and produces distinct ultrastructural changes in the dying cells (Fig. 5C). The lack of dependence on cathepsin B or caspase-1, together with the unique ultrastructural features, lack of dependence on Bax or Bak, and the previously reported lack of dependence on executioner caspases, TNF-α, Toll signals and free radicals of oxygen or nitrogen [5] demonstrate that the macrophage cell death mode induced by virulent Mtb is unlike any previously reported infection-induced cell death. The association of LMP with Mtb cytotoxicity yet no evident requirement for cathepsins directed our attention to other lysosomal hydrolases.

### Mtb-induced membrane damage is associated with phospholipid degradation

**Figure 6:**
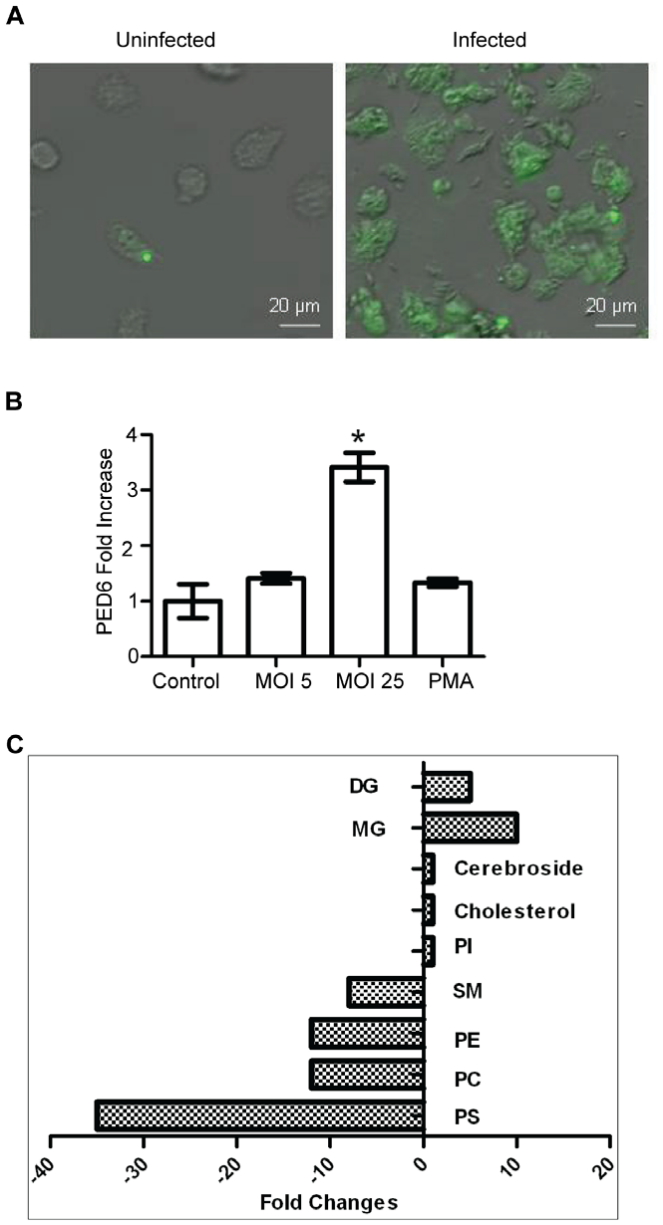
Increased lipase activity in Mtb-infected macrophages. (A) Macrophages loaded with liposomal PED-6 were challenged with Mtb (3 h) and then examined by fluorescence microscopy. Bar  = 20 µm. (B) Macrophages loaded with liposomal PED-6 were cultured in control medium, infected with Mtb, or treated with 1 µmol PMA. Cell lysates were prepared and fluorescence intensity measured by fluorometry was calculated as fold increase relative to uninfected cells and normalized for autofluorescence. (*p<0.05; error bars ± SD). (C) Macrophages were infected with Mtb or maintained in control medium for 6 h and then lysates were prepared for HPLC/MS analysis of total lipid content. Data are expressed as fold change of infected cells compared to uninfected cells. DG, diacylglycerol; MG, monoacylglycerol; PI, phosphatidylinositol; SM, sphingomyelin; PE, phosphatidylethanolamine; PC, phosphatidylcholine; PS, phosphatidylserine.

Widespread membrane damage and Bax/Bak-independent MOMP suggested that lipases might participate in Mtb cytotoxicity. To investigate that question we developed a live cell assay using a PED-6 probe delivered in liposomes. PED-6 is a glycerophosphoethanolamine with a BODIPY dye-labeled acyl chain at the sn2 position and a dinitrophenyl quencher-modified head group. Cleavage of the acyl chains or the head group unquenches BODIPY fluorescence. Uninfected macrophages loaded with PED-6 and examined by microscopy showed minimal fluorescence, while diffuse intracellular fluorescence was observed in Mtb-infected macrophages (Fig. 6A). The microscopy results correlated with total fluorescence of cell lysates measured by fluorometry and was observed in cells infected with a lethal dose of Mtb (MOI 25). PED-6 fluorescence was not detected in cells challenged with Mtb at MOI 5 or following stimulation with phorbol myristate acetate (PMA) to activate lipases normally present in the cytosol that participate in signal transduction such as cytosolic phospholipase A_2_ (Fig. 6B).

Increased lipase activity in Mtb-infected macrophages was confirmed by high performance liquid chromatography and mass spectroscopy (HPLC/MS). Compared to uninfected cells, the total lipid composition of macrophages infected for 6 h with Mtb showed 10 to 35-fold reduction of PS, phosphatidylcholine and phosphatidylethanolamine content with a corresponding 5 to 10-fold increase in monoacylglycerol and diacylglycerol (Fig. 6C). There was no reduction in phosphatidylinositol or cholesterol, but infection also resulted in a 10-fold reduction of sphingomyelin. The shift in lipid composition is consistent with the action of phospholipases acting at the sn1 and sn2 positions as well as phosphatase and sphingomyelinase activities. The HPLC/MS data imply that multiple different lipase activities are unleashed to act on membrane phospholipids in Mtb-infected cells, catabolizing certain phospholipid species to acylglycerides. While mycobacterial lipases could conceivably mediate these effects, the range and potency of the lipase activities unleashed after infection were seen as more likely mediated by lysosomal lipases.

### Lysosomal lipases contribute to Mtb-induced mitochondrial injury and cell death

**Figure 7:**
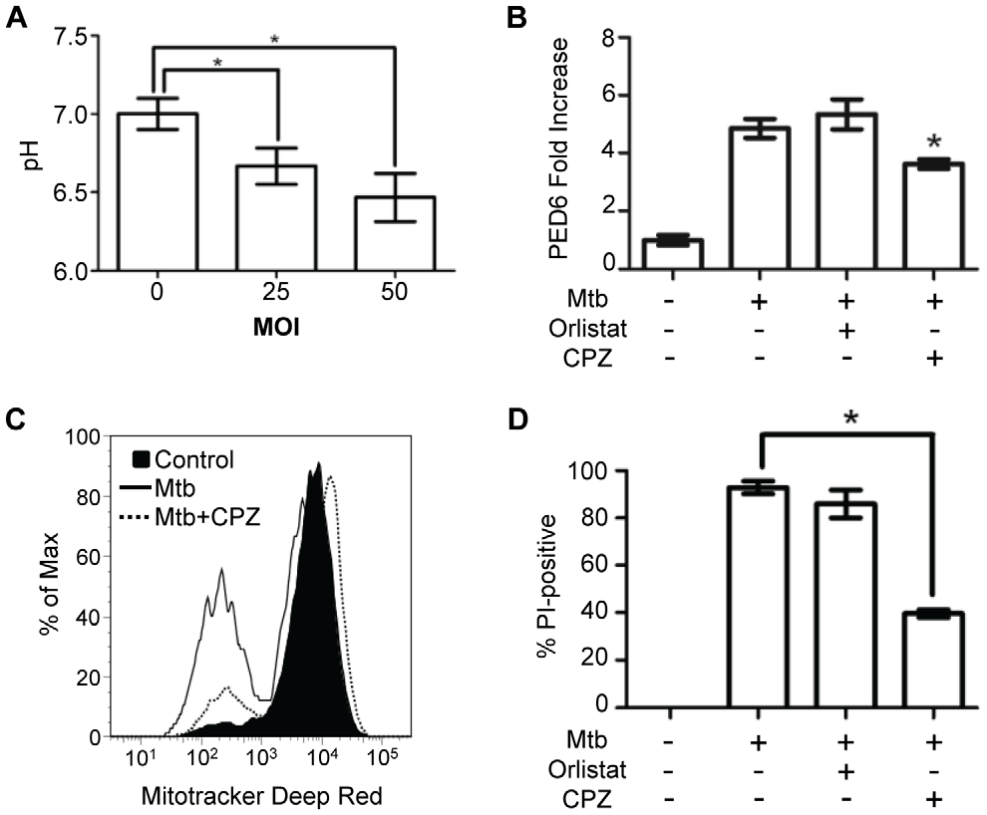
Lysosomal lipases participate in Mtb-induced cytolysis. (A) Macrophages were infected with Mtb at the indicated MOI for 150 min and then cytosolic pH was measured by using the dual emission fluorescent dye SNARF-1 (*P<0.05). (B) Macrophages loaded with liposomal PED-6 were pretreated with control buffer, CPZ or orlistat and then infected with Mtb. Fluorescence intensity in cell lysates was calculated as fold increase relative to uninfected cells and normalized for autofluorescence (*P<0.05). (C) Macrophages with or without CPZ pretreatment were infected with Mtb (3 h) and probed with Mitotracker Deep Red. Histograms are representative of three independent experiments. (D) Macrophages were treated with CPZ, orlistat or control buffer prior to Mtb infection (18 h). Cell death was measured by PI incorporation (*P<0.05).

The HPLC/MS analysis suggested that lipases participate in the catabolism of membrane phospholipids in Mtb-infected macrophages. Lysosomal hydrolases, including enzymes with phospholipase, phosphatase, ceramidase and sphingomyelinase activities [27], [28], are involved in normal recycling of membrane lipids. The identity of all lysosome-associated lipases and their regulation have yet to be defined, although some are known to have low pH optima. In this regard, we found that cytoplasmic pH falls during the course of Mtb-induced cytolysis (Fig. 7A). Chlorpromazine (CPZ) was previously shown to inhibit lysosomal phospholipase and triglyceride lipase activities [29] so we tested its capacity to rescue infected macrophages. The increased cytosolic lipase activity found in infected macrophages was partially blocked by CPZ but not by the pancreatic lipase inhibitor orlistat (Fig. 7B). CPZ exhibited more potent inhibition of Mtb-induced mitochondrial injury (Fig. 7C) and cell death (Fig. 7D). While CPZ possesses antimicrobial activity against Mtb [30], the concentration used (10 µM) was below the minimum inhibitory concentration [31]. Also arguing against any confounding effect of antimicrobial activity in our experiments, CPZ failed to prevent cytosolic translocation of cathepsin B in infected macrophages (Fig. S8) whereas the induction of LMP requires the presence of viable intracellular bacilli. The finding that CPZ did not prevent LMP indicated that its protective effect lies downstream of that early step in Mtb cytotoxicity. Preservation of cell viability despite ongoing LMP may explain why cytosolic translocation of cathepsin B was greater in Mtb-infected macrophages treated with CPZ than untreated, infected macrophages. Considering the TEM and SEM images, the cytochrome *c* staining and cationic dye release studies, the liposomal PED-6 assay, the lipidomic analysis and the effects of CPZ, we conclude that lysosomal lipases contribute to the membrane damage that characterizes macrophage cell death induced by Mtb. Release of lysosomal lipases might not fully account for these changes since enzymatic digestion of intact lipid bilayers, as occurs in lysosomes, requires unique conditions and carrier proteins [32]. This suggests that as yet uncharacterized membrane destabilizing agents may facilitate the enzymatic catabolism of cell membrane phospholipids in Mtb-infected macrophages.

### Mtb induces LMP in a PhoPR-dependent manner

**Figure 8:**
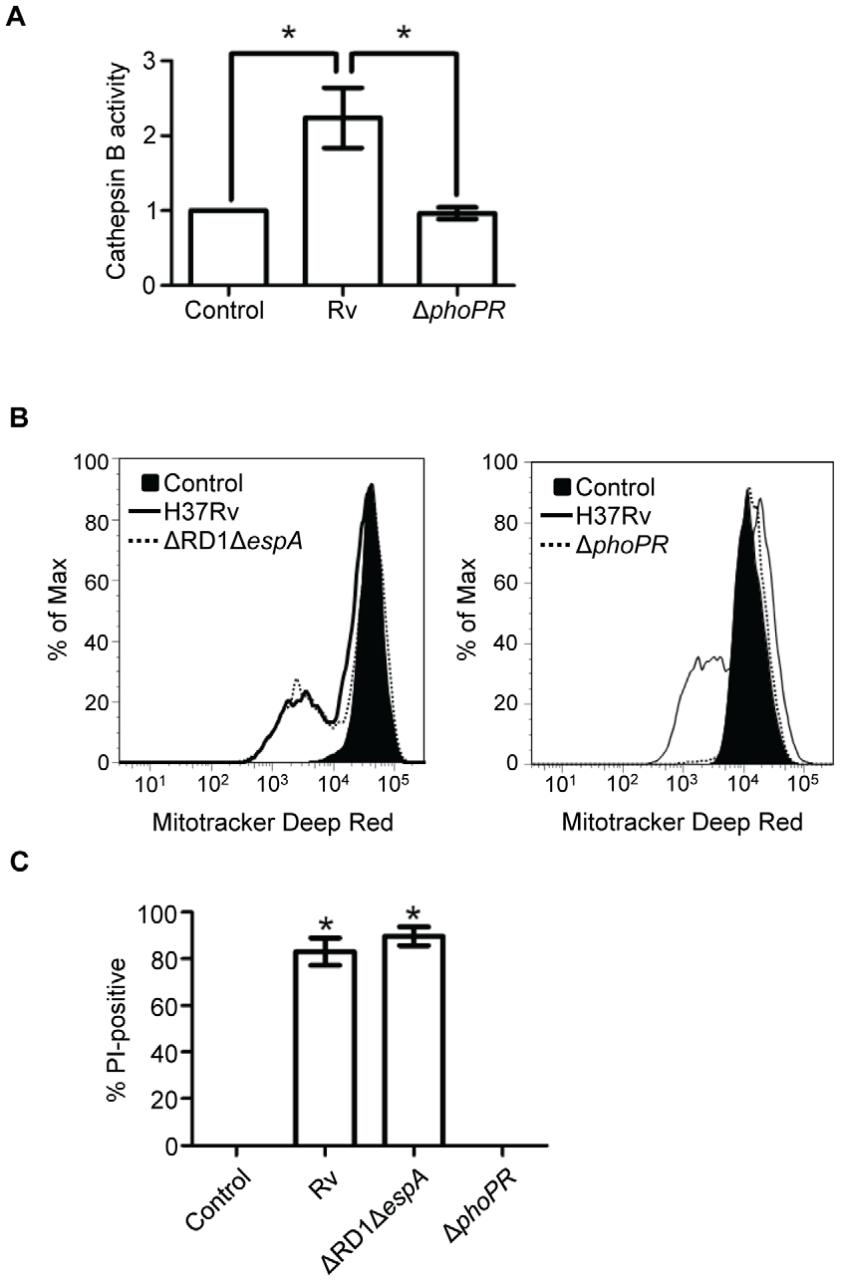
The PhoPR two component system is required for LMP, lipase activation, mitochondrial injury and death of Mtb-infected macrophages. (A) Macrophages were challenged with RvΔ*phoPR* or parental H37Rv and LMP was measured by cytosolic translocation of cathepsin B 2 h later. Results are expressed as mean fold increase of cytosolic cathepsin B activity ± SEM. * *P*<0.05. (B) Mitochondrial injury (ΔΨ_m_ dissipation) was measured in macrophages challenged with H37Rv as compared to RvΔRD1Δ*espA* (*left panel*) or RvΔ*phoPR* (*right panel*) using Mitotracker Deep Red. Histograms are representative of three independent experiments. (C) Cell death assessed by PI incorporation was measured in uninfected macrophages and macrophages challenged with H37Rv, RvΔRD1Δ*espA*, or RvΔ*phoPR* (18 h). A representative experiment of three performed is depicted. * *P*<0.05 comparing cells infected with H37Rv or RvΔRD1Δ*espA* to uninfected cells or cells infected with RvΔ*phoPR*. Error bars, ± SD.

Having excluded a requirement for the ESX1 system in cytolytic activity we concluded that other Mtb genes must be responsible for infection-induced LMP in our system. Since viable intracellular bacteria were required to induce cell death, we hypothesized that altered bacterial gene expression in response to the intracellular milieu may be required. Much of the early transcriptional response of the bacterium to this environment is controlled by the PhoPR two component regulatory signal system [33]. An Mtb *phoP* mutant (SO2) was reported by Ferrer et al. [7] to be attenuated in THP-1 monocytic cells due to an impaired capacity to block phagolysosome fusion. As with BCG, the inability of SO2 to grow in macrophages would prevent it from reaching a lethal intracellular burden after low MOI challenge thereby making it appear non-cytotoxic even if it retained the potential to kill. However, SO2 is capable of replication in fibroblasts and does not cause cytotoxicity in those cells [34]. To explore the possible involvement of the PhoPR regulon in macrophage cytotoxicity we generated a mutant strain (RvΔ*phoPR*) and compared it with wildtype H37Rv in macrophages challenged at MOI 25. Compared to wildtype H37Rv, the PhoPR deficient strain failed to induce LMP (Fig. 8A) or mitochondrial injury (Fig. 8B) and it was severely attenuated in its ability to induce macrophage cell death (Fig. 8C). Consistent with the reported phenotype of SO2, RvΔ*phoPR* was not impaired in its ability to enter macrophages, with a trend for intracellular numbers greater than H37Rv after challenge at equivalent MOI (Fig. S9). This is consistent with the reported increased binding of SO2 to macrophages and confirms the attenuation of RvΔ*phoPR* for cytotoxicity at high intracellular bacillary load.

## Discussion

We previously described caspase-independent macrophage cell death induced by Mtb when the intracellular load exceeds ∼20 bacilli [5]. In that report, infected macrophages were shown to exhibit apoptotic features of nuclear condensation and PS translocation but rapidly progressed to necrosis. In the present study we have further elucidated the mode and mechanism of Mtb-induced cell death. Infected macrophages show an atypical combination of apoptotic and necrotic ultrastructural features notable for widespread membrane damage. LMP is an early event that is followed by mitochondrial outer and then inner membrane permeabilization which is independent of Bax and Bak. Mitochondrial injury in the absence of these Bcl-2 family members indicates that Mtb-induced cell death does not operate through the classical intrinsic apoptosis pathway. Dissipation of ΔΨm, depletion of intracellular ATP and a drop in cytosolic pH are followed by widespread loss of membrane integrity observed by electron microscopy. This is accompanied by catabolism of several phospholipid species that are integral constituents of lipid bilayers. Factors required for pyroptosis and pyronecrosis (cathepsin B and caspase-1, respectively) are dispensable for Mtb cytotoxicity. The constellation of findings shown here and in our previous report is unique among infection-induced cell death modes.

The early appearance of LMP in the chain of events resulting in macrophage cell death, and the loss of cytotoxicity in bacilli incapable of causing LMP, implicate involvement of a lysosomal death pathway. Lysosomal cell death, which results from relocation of hydrolases into the cytosol, has been linked to a wide variety of pathological conditions including certain infections but not Mtb [35], [36]. Cells dying by this process may exhibit primarily apoptotic or primarily necrotic characteristics depending on the nature and extent of injury to the limiting membrane of the lysosome [37]. The biochemical pathways mediating lysosomal death vary considerably in different cell types and with conditions of induction, including examples that are caspase-dependent or caspase-independent [35]. Lysosomal cell death has been most frequently attributed to cytosolic translocation of cathepsins, particularly cathepsins B, L, S and D. We previously reported partial protection from Mtb cytotoxicity by chemical inhibitors of cathepsins B and L, but not cathepsin D [5]. However, the effects were modest and limited by direct cytotoxicity of these agents. Subsequent experiments revealed that these agents also partially inhibit phagocytosis of Mtb (Fig. S10) which would promote macrophage survival by decreasing intracellular bacillary load at a given MOI. Our current studies with BafA1, cathepsin B silencing and cathepsin L null macrophages (Fig. 4) provide additional evidence that Mtb-induced cell death does not depend on cathepsins. Instead, the ultrastructural evidence of widespread membrane damage, the degradation of multiple phospholipid species and the evidence of Bax/Bak independent MOMP and subsequent loss of ΔΨ_m_ are all consistent with membrane attack mediated by lysosomal lipases, possibly with a contribution of mycobacterial lipases and/or membrane-destabilizing detergent-like molecules. Bioenergetic collapse in this setting could be lethal but cell death appears inevitable regardless of mitochondrial function, given the catastrophic disruption of nuclear and plasma membranes. This model of Mtb-induced macrophage cell death is supported by the appearance of widespread lipase activity in infected macrophages revealed using a liposomal PED-6 probe and by the protective effects of CPZ which acts downstream of LMP. It is also consistent with previously reported effects of lysosomal lipases on mitochondrial membrane integrity [20]. Our model of membrane injury may explain with the observations of Divangahi et al. [38] that the fate of Mtb-infected macrophages depends in part on their capacity for membrane repair.

In the conditions of our experiments, only viable Mtb are capable of killing macrophages. This indicates that one or more bacterial gene products are essential for the LMP that results in cell death. Evidence supporting this conclusion includes the capacity of macrophages to survive with a high intracellular load of *M. smegmatis* as we reported [5] or *M. leprae* as reported by others [39]. While the ESX1 secretion system and ESAT-6 have been implicated in membrane injury and LMP, and are clearly associated with Mtb virulence, we found that they are dispensable for macrophage cytolysis at high MOI. Having eliminated a requirement for the ESX1 system we next tested RvΔ*phoPR* based on the in vivo attenuation of this strain [40], [41], the role of the PhoPR two-component system in altering bacterial gene expression upon phagocytosis, and the recent report that fibroblasts permit bacillary replication and survive a high intracellular burden of the PhoPR-deficient strain SO2 [34]. We found that that the RvΔ*phoPR* mutant was significantly impaired in its ability to induce LMP, mitochondrial injury and macrophage cell death after challenge at MOI 25. The attenuation of cytotoxicity could not be attributed to any failure of bacilli to enter macrophages. Indeed, the intracellular burden of RvΔ*phoPR* was greater than that of the parental strain H37Rv after challenge at similar MOI. These results indicate that one or more essential mediator(s) of proximal events in macrophage cytotoxicity lie within the PhoPR regulon. Which Mtb gene(s) are responsible for promoting LMP and cytolysis from among the 54 so far known to be regulated by PhoPR remains to be determined. It is worth noting that 7 genes of this regulon are predicted to be involved in lipid metabolism [41], [42]. While the preponderance of data suggest that host lysosomal lipases are the executioners of Mtb-infected cells, our studies do not exclude a role for mycobacterial lipases or other bacterial products such as lipid carrier proteins or lipids with detergent properties that could disrupt membranes to cause LMP and/or facilitate lipolytic membrane attack.

We delivered attenuated mycobacteria (BCG, RvΔRD1Δ*espA* and RvΔ*phoPR*) in higher intracellular numbers than could be achieved by their limited capacity for replication in macrophages after low MOI challenge. Our in vivo data (Fig. S2) unequivocally support the relevance of high bacillary load to pulmonary TB disease. High MOI challenge in vitro makes it possible to distinguish between reduced cytopathicity based on the loss of microbial mediators of host cell death versus reduced capacity for bacteria introduced at low MOI to grow to a number capable of killing the host cell. Our experiments model events that occur when macrophages engulf a small number of virulent bacilli that subsequently kill the host cell after a period of intracellular replication. This concept was directly tested in our published MOI/cell death dose-response study [5] and in detailed experiments by Park et al. [4] where macrophages were challenged at MOI 5 with several Mtb strains differing in virulence. The most virulent strains grew rapidly over 6 days and killed their host cells after reaching an intracellular burden similar to the cytoxic threshold we found with direct high MOI challenge. Park et al. also showed that pretreatment with IFN-γ suppressed Mtb replication and preserved macrophage viability at day 6 post-infection. This confirmed that cytotoxicity depends on bacterial burden and is not an intrinsic property of certain virulent strains unrelated to intracellular bacillary load.

Lung macrophages provide an environment required by the facultative intracellular pathogen Mtb to establish infection in a new host [43]. Successive rounds of intracellular bacillary replication followed by host cell lysis, invasion of naïve macrophages and resumption of intracellular replication are essential steps for the exponential increase in lung bacillary burden following aerosol infection. This cycle is interrupted by the expression of adaptive immunity. Once IFN-γ is present in sufficient quantity in the lung it activates macrophages to limit Mtb replication and thereby survive with a sublethal load of persistent bacilli. The mode and rate of infection-induced macrophage cell death may be determining factors in the pathogenesis TB. The known anti-apoptotic functions of virulent Mtb could result in a more indolent disease like leprosy without a means for bacilli to eventually exit the macrophage. The death mode that we describe in macrophages infected with virulent Mtb has several features consistent with its proposed role in TB pathogenesis: 1, it is induced only after intracellular bacilli have accumulated to a high number; 2, bacilli are released to the extracellular space without confinement in apoptotic vesicles; 3, macrophage cell death is not accompanied by a significant loss of bacillary viability; 4, the pro-inflammatory nature of macrophage necrosis might aid in the recruitment of naïve phagocytes to host subsequent rounds of infection; and 5, macrophage death and unchecked lipolysis might help shape a nutrient milieu in necrotic TB lesions supporting extracellular bacterial survival and disease transmission.

## Materials and Methods

### Ethics statement

Experiments with animals were conducted according to the National Institutes of Health guidelines for housing and care of laboratory animals under protocols approved by the Institutional Animal Care and Use Committee (A-1429) and the Institutional Biosafety Committee (I-161) at The University of Massachusetts Medical School.

### Bacterial strains and reagents

Bacterial strains used in this study were wildtype Mtb Erdman, Mtb H37Rv and *M. bovis* BCG, the H37Rv mutant strains mc^2^6020 (Δ*lysA* Δ*panCD* double auxotroph) [8], ΔRD1*ΔespA* [9] and Δ*phoPR* as well as *Salmonella typhimurium* strain SL1344. Unless otherwise indicated, experiments were performed with Erdman or mc^2^6020. These two had equivalent cytolytic activity at MOI 25; mc^2^6020 was used when BSL-2 containment was necessary for the performance of particular assays. The Mtb Δ*phoPR* mutant was generated by allelic exchange of the *phoPR* coding region using positive selection for hyg resistance and sucrose counter-selection [10]. The *phoPR* deletion in this mutant corresponds to the H37Rv genomic coordinates 851640 to 853817 (*phoPR* CDS. 851608 to 853853).

The pancreatic lipase inhibitor orlistat, the cyclophilin D inhibitor cyclosporine A, the vesicular proton pump ATPase inhibitor bafilomycin A1, the lysosomal lipase inhibitor chlorpromazine and neutral red were purchased from Sigma-Aldrich. The protein kinase inhibitor staurosporine (Sigma-Aldrich) was used as a positive control for apoptosis. Staining dyes including 4′,6-diamidino-2-phenylindole, dihydrochloride (DAPI), PI, MitoTracker Red CMXRos, MitoTracker Deep Red FM, PED-6 (*N*-((6-(2,4-dinitrophenyl)amino)hexanoyl)-2-(4,4-difluoro-5,7-dimethyl-4-bora-3a,4a-diaza-*s-*indacene-3-pentanoyl)-1-hexadecanoyl-*sn-*glycero-3-phosphoethanolamine, triethylammonium salt were purchased from Invitrogen. Lipid components for liposome generation include 1,2-dipalmitoyl-*sn*-glycero-3-phosphocholine (DPPC), 1,2-dipalmitoyl-*sn*-glycero-3-[phospho-L-serine] (DPPS), 3β-[N-(N',N'-dimethylaminoethane)-carbamoyl]cholesterol hydrochloride (DC-cholesterol) were purchased from Avanti.

### Cells

Bone marrow derived macrophages were generated from wildtype C57/BL6 mice or *Bak1* null mice with a floxed *Bax* allele (B6;129-*Bax^tm2Sjk^ Bak1^tm1Thsn^*/J) and mice with targeted mutation of caspase-1 (NOD.129S2(B6)-*Casp1^tm1Sesh^*/LtJ) purchased from the Jackson Laboratory. Macrophages were prepared by culturing bone marrow cells in DMEM medium (Gibco) supplemented with 10% L929 conditioned medium, 10% FBS (BioWhittaker), 100 units/ml of penicillin, 100 mg/ml of streptomycin, and 2 mM glutamine (complete DMEM) for 7 days. *Bax* knockout was produced with a retrovirus expressing Cre recombinase. For retrovirus production, EcoPack 293 cells (Clontech) were stably transduced with a vector generated by inserting a Cre recombinase cDNA into pMSCV (Clontech). Macrophages were transduced with the virus on the second day of culture and harvested on the eighth day. Transuded cells were isolated by fluorescence activated cell sorting based on GFP expression.

### Bacterial infections

Bacterial stocks were prepared by growing strains at 37°C with continuous agitation in Middlebrook 7H9 broth supplemented with 10% OADC, 0.2% glycerol and 0.05% Tween 80 or further supplemented with 80 µg/ml lysine and 25 µg/ml pantothenate (for mc^2^6020). Bacteria in log growth phase (OD_600_ 0.5 to 0.7) were washed twice and resuspended with PBS containing 0.05% Tween 80. Bacterial concentration was determined by plating on 7H11 agar with OADC (DIFCO; Beckton Dickinson) and counting colony forming units. Macrophages were plated in 8 well Lab-Tek tissue culture chamber slides (Nalge Nunc International) at 2×10^5^ cells per well for PI/DAPI staining and cytochrome *c* staining, or in 24 well plates at 5×10^5^ cells per well for mitochondrial staining, PED-6 assay, or cathepsin B assay. Cells were infected with Mtb strains at MOI 25 (unless indicated otherwise) in antibiotic-free complete DMEM at 37°C for 3 h and then washed to remove unbound bacteria and further incubated for the indicated times in complete DMEM.

To evaluate the relevance of high burden in vitro infections to events occurring in TB disease in vivo, C57BL/6 mice were infected with Mtb Erdman by aerosol (Glas-Col) set to deliver 100 CFU to the lung. Two mice were sacrificed 24 h after aerosol exposure to confirm the delivered dose of Mtb. Two weeks after aerosol infection mice were euthanized; lung leukocytes were isolated as previously described [11] and then prepared by cytocentrifugation (Thermo Electron Corporation) for Ziehl-Neelsen acid fast stain (BD Biosciences).

The mouse-virulent *S. typhimurium* strain SL1344 was grown in Difco Luria-Bertani (LB) broth or agar. To obtain stationary phase bacteria, LB broth was inoculated with a single colony and was grown with aeration at 37°C for 8 h. The stationary culture was diluted 1,000-fold and grown to late logarithmic phase standing at 37°C for 16 h. Bacteria were washed once with complete media without antibiotics and resuspended in antibiotic-free complete DMEM for infection. Macrophages plated in Lab-Tek chamberslides (Nalge Nunc International) were infected with various doses of *S. typhimurium* and the cell death was measured by neutral red uptake assay 4 h after infection.

### Transmission electron microscopy

Following infection, cells were processed by washing with PBS and fixed with 2% paraformaldehyde (v/v)/2.5% glutaraldehyde (v/v) in 0.1 M Na cacodylate-HCl buffer (pH 7.2) overnight at 4°C. The next day the fixed samples were washed three times in 0.5 M Na cacodylate-HCl buffer (pH 7.0). The cells were then scraped from the wells, collected in a microfuge tube, pelletted and post-fixed 1 h in 1% osmium tetroxide (w/v). Following post-fixation, the pelletted cells were en block-stained (20 min) with 1% aqueous uranyl-acetate (w/v) then washed again in the same buffer and dehydrated through a graded series of ethanol to 100%, transferred through two changes of propylene oxide and then into a mixture of 50% propylene oxide/50% Spurr's resin and left overnight to infiltrate. The following day cell pellets were transferred through three changes of fresh Spurr's resin and then embedded in molds filled with a mixture of Spurr's epoxy resin and polymerized for 48 h at 70°C. The epoxy blocks with then trimmed and ultra-thin sections were cut on a Reichart-Jung ultramicrotome using a diamond knife. Cut sections were mounted on copper support grids and contrasted with lead citrate and uranyl acetate and then examined on a Philips CM 10 transmission electron microscope at 80 Kv accelerating voltage.

### Scanning electron microscopy

Mtb-infected cells were fixed as described above, the entire bottom of the well plates were cut out and dehydrated through a graded series of ethanol to 2 changes of 100% ethanol and critical point dried in liquid CO_2_. The dish bottoms with the dried cells were mounted onto aluminum stubs with silver conductive paste and sputter coated with gold/palladium (4 nm). The specimens were then examined using an FEI Quanta 200 FEG scanning electron microscope.

### Cytochrome *c* staining

Cells were challenged with Mtb for 2 h or 3 h and then washed twice in PBS and fixed with 4% paraformaldehyde for 20 min on ice. Cells were next washed 4 times with PBS and permeabilized with 0.2% Triton dissolved in PBS for 5 min at room temperature. Cells were blocked with 10% FBS in PBS for 2 h and stained with 2 ug/ml of anti-cytochrome *c* (BD Biosciences) in PBS containing BSA 0.5% overnight at 4°C. After washing, cells were stained with 7.5 ug/ml of rhodamine TRITC-labeled donkey anti-mouse IgG (Jackson ImmunoResearch Laboratories) for 2 h at room temperature and then slides were washed and stained with DAPI and mounted with Anti-Fade solution (Invitrogen) for examination by fluorescent microscopy.

### Mitochondrial membrane potential

After a period of infection, cells were incubated with 100 nM of Mitotracker CMXRos or Mitotracker Deep Red in complete media for 20 min. Cells were detached with 0.05% trypsin and 0.02% EDTA for 25 min, washed, and fixed with 1% paraformaldehyde. Flow cytometry was performed on an LSRII flow cytometer (BD Bioscience Pharmingen), 50,000 leukocyte-gated events were collected and data analyzed with FlowJo PC software (TreeStar, Inc.).

### Cytosolic cathepsin B assay

Cytosolic fractions were prepared as described by Foghsgaard et al. [12] Briefly, cells were washed with PBS and incubated with permeabilization solution (digitonin 25 ug/ml, sucrose 250 mM, HEPES 20 mM, KCl 10 mM, MgCl_2_ 1.5 mM, EDTA 1 mM, EGTA 1 mM, Pefablock 1 mM, pH 7.4) for 5 min. The concentration and duration of incubation with permeabilization solution was based on the maximum release of lactate dehydrogenase (LDH). Cathepsin B activity was measured by commercial assay (BioVision) according to manufacturer's protocol. Equal volumes of cytosolic fraction and reaction buffer from the assay kit were mixed along with cathepsin B substrate Ac-RR-AFC and incubated for 1 h at 37°C. The fluorescence was measured in a fluorometer equipped with a 400-nm excitation filter and 505-nm emission filter. LDH levels were measured to normalize cathepsin B activity. Data are expressed as fold increase in cathepsin B activity relative to control cells divided by fold increase in LDH relative to control cells.

### Cathepsin B silencing

27mer RNA oligonucleotides for silencing mouse cathepsin B were purchased from IDT Technologies. The sense strand of the siRNA duplex consisted of a 25 nt target sequence (5′-cccuagacuagugcgguuugaagtg-3′) and the antisense strand was composed of nucleotides complementary to the target sequence and the AG 3′ overhang (5′-cacuucaaaccgcacuagucuagggag-3′). siRNA for luciferase was used as a control. Two hundred thousand macrophages on a Lab-Tek chamberslides were transfected with 10 uM of siRNA using Oligofectamine (Invitrogen) according to the manufacturer's instructions. The efficacy of cathepsin B silencing was confirmed by measuring the reduction of total cellular enzymatic activity (Fig. S1).

### Liposomal PED-6 lipase assay

Lipase activity was measured with a fluorescent PED-6 probe delivered in liposomes composed of 0.05 µM PED-6, 1.4 µM DPPC, 1 µM DPPS and 1 µM DC-cholesterol mixed in chloroform and dried in a Buchi rotavapor (BUCHI Labortechnic AG). Thin layers of dried mixed lipids were dispersed in 1 ml PBS to make multilamellar vesicles. Multilamellar vesicles were extruded with Liposofast (Avestin) through 0.1 µm pore size polycarbonate filters to make liposomes of uniform size. Twenty ul of liposome solution was added to Mtb-infected macrophage cultures, incubated for 16 h, washed twice with PBS and then lysed with 1% SDS PBS for 10 min. Lysates were centrifuged and supernatant were collected to measure fluorescence at excitation 480 nm and emission 530 nm.

### HPLC-MS lipid analysis

Infected and uninfected cells were detached with trypsin-EDTA and washed with PBS. Cell pellets were resuspended in 100 µl HPLC grade water and then mixed with 20 vol of chloroform:methanol (2∶1, v:v). Total lipid profiles were determined by atmospheric pressure chemical ionization HPLC-MS. The lipids were quantified by determining the ratio of the analyte chromatographic peak area to the internal standard peak area for each lipid and comparing that to the ratio for the same lipid analyzed separately in a mixture of 20 standard lipids.

### Determination of cytosolic pH

Cytosolic pH was measured using dual emission fluorescent dye, SNARF-1 (Invitrogen). Macrophages infected with Mtb for 150 min were stained with SNARF-1 according to the manufacturer's protocol. Briefly, cells were rinsed with serum-free DMEM and incubated with pre-warmed serum-free DMEM containing 10 µM of SNARF-1 for 30 min at 37°C. Cells were then detached from plates with 0.05% trypsin-EDTA, washed with PBS, and resuspended in a pH 7 buffer consisting of 1∶1 ratio of KH_2_PO_4_ buffer (110 mM KH_2_PO_4_ and 20 mM NaCl) and K_2_HPO_4_ buffer (135 mM K_2_HPO_4_ and 20 mM NaCl). To produce a standard pH curve, uninfected cells were stained with SNARF-1 and resuspended in buffers of various pH (different ratios of KH_2_PO_4_ buffer and K_2_HPO_4_ buffer) containing 10 µM nigericin. For flow cytometric analysis, cells were excited by a 488 nm laser and fluorescence emission was monitored at 575 nm and 695 nm using a FACSAria (BD) located in a BSL 3 facility. The ratio of the dual emissions was calculated by dividing the mean fluorescent intensity at 575 nm by the one at 695 nm. The linear equation obtained from the standard curve was used to calculate the cytosolic pH.

### Statistics

Results are expressed as mean ± SD or SEM of data obtained in independent experiments. Differences between groups were assessed by a one-way ANOVA or student's *t* test using GraphPad Prism. Values of *p*<0.05 were considered statistically significant.

## Supporting Information

Figure S1 **Validation of cathepsin B silencing.** Cells were transfected with cathepsin B siRNA or luciferase siRNA (control siRNA) for 4 days as described in Material and Methods. The total cathepsin activity of cells transfected with cathepsin B siRNA is expressed as the % of activity measured in cells transfected with control siRNA.
(TIF)

Figure S2 **High intracellular bacillary load in lung macrophages following aerosol Mtb infection.** C57BL/6 mice were infected with Mtb Erdman in a Glas-Col Inhalation Exposure System set to deliver 100 CFU to the lungs. Lung leukocytes were harvested 2 wk post-infection and then stained for acid fast bacilli. Some heavily infected macrophages appeared to be in the process of dying and releasing large numbers of bacilli (F) reminiscent of the SEM images obtained from macrophages infected with Mtb in vivo (Fig. S3B). Magnification, X400.
(TIF)

Figure S3 **Degradation of lipid bilayers following Mtb infection permits escape of bacilli through damaged plasma membranes.** (A) TEM images of macrophages infected with Mtb (6 h) showing widespread membrane damage. Structural changes include disruption of phagosomal (*arrow* 1 and 2) and nuclear (*arrow* 3) membranes. Bar  = 500 nm. (B) SEM image of macrophages disorging Mtb bacilli 3 h post-infection. Bar  = 10 µm.
(TIF)

Figure S4 **Mitochondrial injury in infected macrophages.** (A) Macrophages were infected with BCG (3 h) and then stained with Mitotracker CMXRos and DAPI for examination by fluorescence microscopy. White arrows indicate cells with loss of Δψ_m_ demonstrated by relase of fluorescent dye from mitochondria (X, 400). (B) Macrophages plated in 24-well plates at 5×10^5^ per well were infected with BCG or Mtb Erdman for 3 h. Cells were washed with PBS twice and the cellular ATP level after was measured with ApoSENSOR™ ADP/ATP Ratio Assay kit (BioVision) following the manufacturer's protocol. (C) Macrophages were challenged with viable or heat-killed (*HK*) Mtb (3 h) and then probed with Mitotracker CMXRos for analysis by flow cytometry.
(TIF)

Figure S5 **CsA blocks** ***m*** **-chlorophenylhydrazone-induced mitochondrial PT pore formation.** Macrophages were treated with 20 uM of *m*-chlorophenylhydrazone (CCCP) in the presence or absence of of 5 uM CsA for 24 h and then stained with 10 uM of rhodamine 123 (R123) for 15 min. Cells were washed and the R123 fluorescence was assessed by flow cytometry. CCCP-induced mitochondrial PT pore formation as evidenced by low R123 retention by mitochondria was reversed by CsA treatment.
(TIF)

Figure S6 **Induction of LMP by Mtb and BCG.** (A) Macrophages were infected with Mtb (2 h) and then cytosolic extracts were prepared for measurement of cathepsin B activity indicative of LMP. Results are expressed as the fold increase of cathepsin B activity relative to uninfected (*control*) cells, normalized by the fold increase in LDH compared to uninfected cells (*P<0.05; error bars, ± SD). (B) Cytosolic cathepsin B activity in macrophages challenged with BCG or Mtb (*P<0.05).
(TIF)

Figure S7 **Bafilomycin A inhibits LMP and chloroquine-induced lysosomal cell death.** (A) BafA1 abrogates acidification of lysosome leading to loss of acridine orange (*AO*). Cells were loaded with 5 µg/ml of AO in RPMI 1640 medium without serum for 15 min at 37°C. Cells were then treated with BafA1 for 3 h and red fluorescence (lysosomal AO) and green fluorescence (cytosolic AO) intensity was measured by flow cytometry. (B) BafA1 and concanamycin A (ConcA), both inhibitors for vacuolar-type ATPase, prevent cathepsin B activation. Macrophages were treated with 50 nM of BafA1 or ConcA for 1, 3, or 5 h, and total cellular cathepsin B activity was measured. Results are expressed as the mean cathepsin B activity ± SD. (C) BafA1 prevents chloroquine-induced lysosomal cell death. Macrophages treated as indicated with 50 nM of BafA1 and/or chloroquine at 100 µM overnight. Cell death was measured by PI-staining, expressed as the mean % PI-positive cells ± SD.
(TIF)

Figure S8 **Chlorpromazine does not prevent LMP in Mtb-infected cells.** Macrophages were pretreated with CPZ (10 µM) and challenged with with Mtb (2 h) in the presence of CPZ (10 µM). Cytosolic extracts were prepared for measurement of cathepsin B activity. Data are represented as mean fold increase of cytosolic cathepsin B (normalized to LDH as an indicator of cytosolic content) ± SEM (*p<0.05).
(TIF)

Figure S9 **Macrophages engulf RvΔ** ***phoPR*** **more avidly than H37Rv.** (A) Macrophages were challenged with H37Rv (*right panels*) or RvΔ*phoPR* (*left panels*) at MOI 10. Unbound bacteria were removed by washing 3 h later and then slides were stained for acid fast bacilli. Magnification, X400. (B) The number of bacteria associated with macrophages was counted in 3 randomly selected fields in each of 2 Labtek chamberslide wells (∼300 macrophages per field) for macrophages challenged with H37Rv (*open bars*) or RvΔ*phoPR* (*filled bars*). Results are displayed as the mean % macrophages infected with each designated range of bacterial number per chamberslide ± SD.
(TIF)

Figure S10 **Cathepsin inhibitors reduce phagocytosis of Mtb by macrophages.** Macrophages were pretreated with the cathepsin B inhibitor Ca-074-Me 40 µM plus the cathepsin L inhibitor Z-LLY-FMK 5 µM (*CALLY*) or control buffer and then challenged with Mtb H37Rv expressing red fluorescent protein (MOI 25, 3 hr). Phagocytosis of fluorescent bacteria was assesed by flow cytometry.
(TIF)
