# Supplementary figures and images for: Mycobacterium tuberculosis Induces an Atypical Cell Death Mode to Escape from Infected Macrophages
Source: PLoS One. 2011 Mar 31;6(3):e18367. doi: 10.1371/journal.pone.0018367 (PMC3069075; doi:10.1371/journal.pone.0018367)

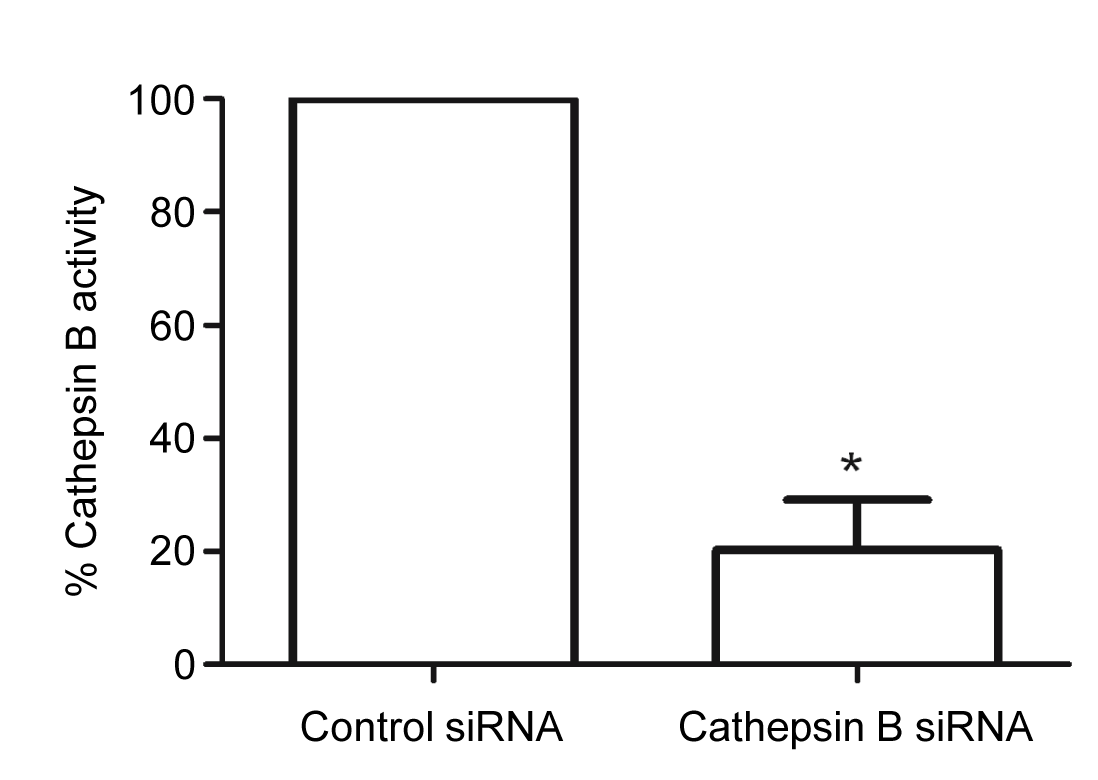

Supplement: Figure S1 — Validation of cathepsin B silencing. Cells were transfected with cathepsin B siRNA or luciferase siRNA (control siRNA) for 4 days as described in Material and Methods. The total cathepsin activity of cells transfected with cathepsin B siRNA is expressed as the % of activity measured in cells transfected with control siRNA. (TIF) [file pone.0018367.s001.tif]

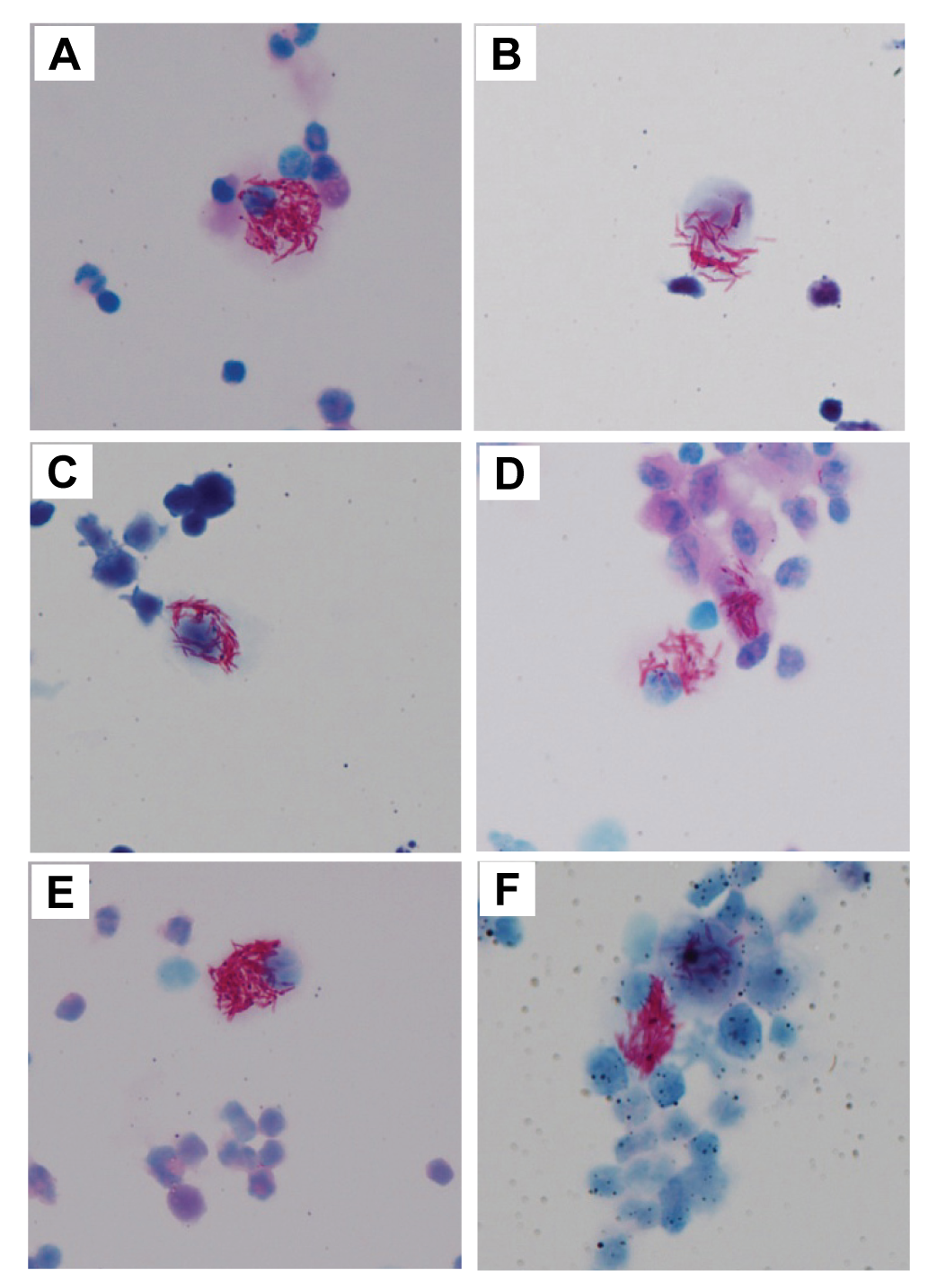

Supplement: Figure S2 — High intracellular bacillary load in lung macrophages following aerosol Mtb infection. C57BL/6 mice were infected with Mtb Erdman in a Glas-Col Inhalation Exposure System set to deliver 100 CFU to the lungs. Lung leukocytes were harvested 2 wk post-infection and then stained for acid fast bacilli. Some heavily infected macrophages appeared to be in the process of dying and releasing large numbers of bacilli (F) reminiscent of the SEM images obtained from macrophages infected with Mtb in vivo (Fig. S3B). Magnification, X400. (TIF) [file pone.0018367.s002.tif]

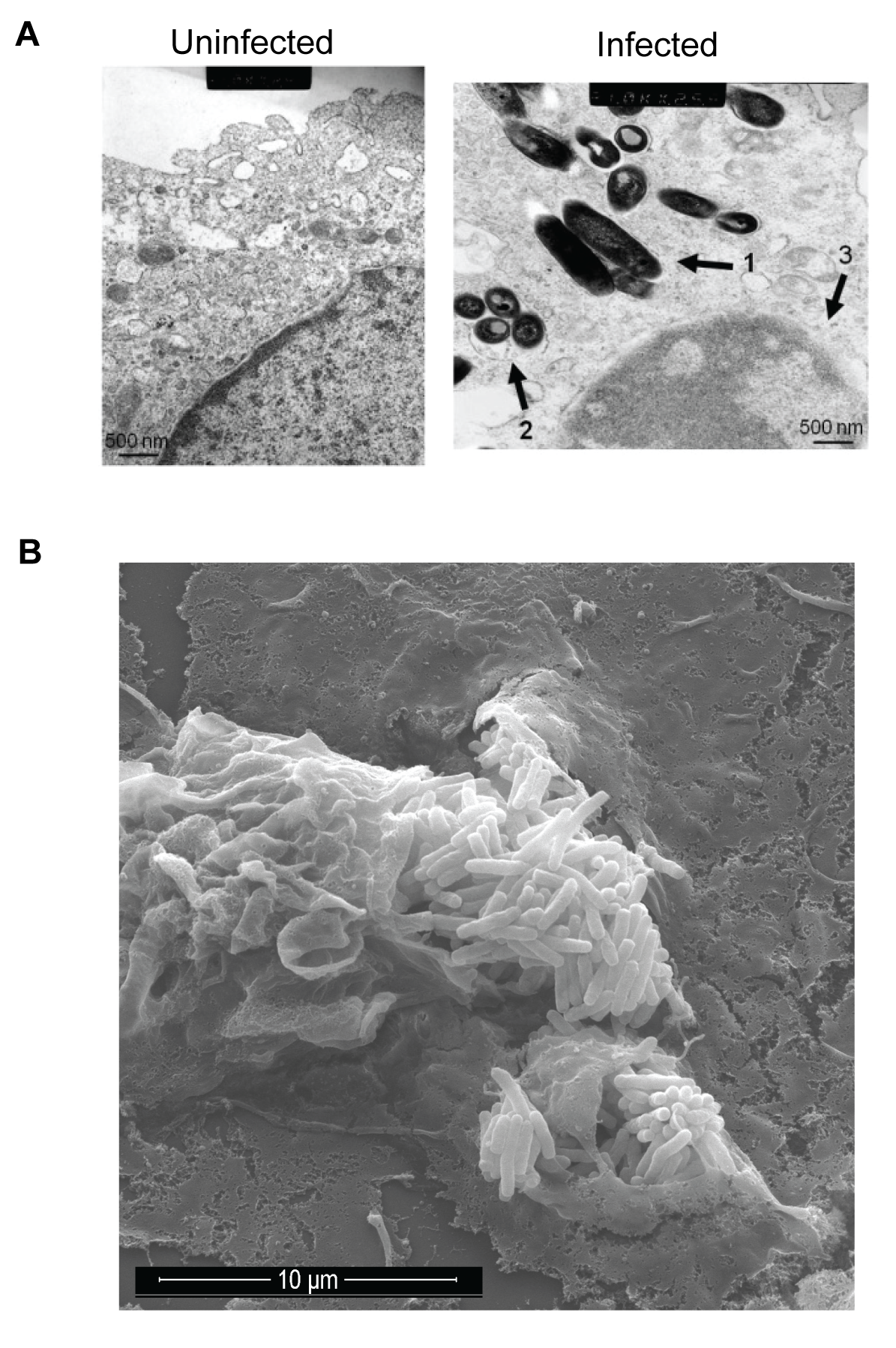

Supplement: Figure S3 — Degradation of lipid bilayers following Mtb infection permits escape of bacilli through damaged plasma membranes. (A) TEM images of macrophages infected with Mtb (6 h) showing widespread membrane damage. Structural changes include disruption of phagosomal (arrow 1 and 2) and nuclear (arrow 3) membranes. Bar = 500 nm. (B) SEM image of macrophages disorging Mtb bacilli 3 h post-infection. Bar = 10 µm. (TIF) [file pone.0018367.s003.tif]

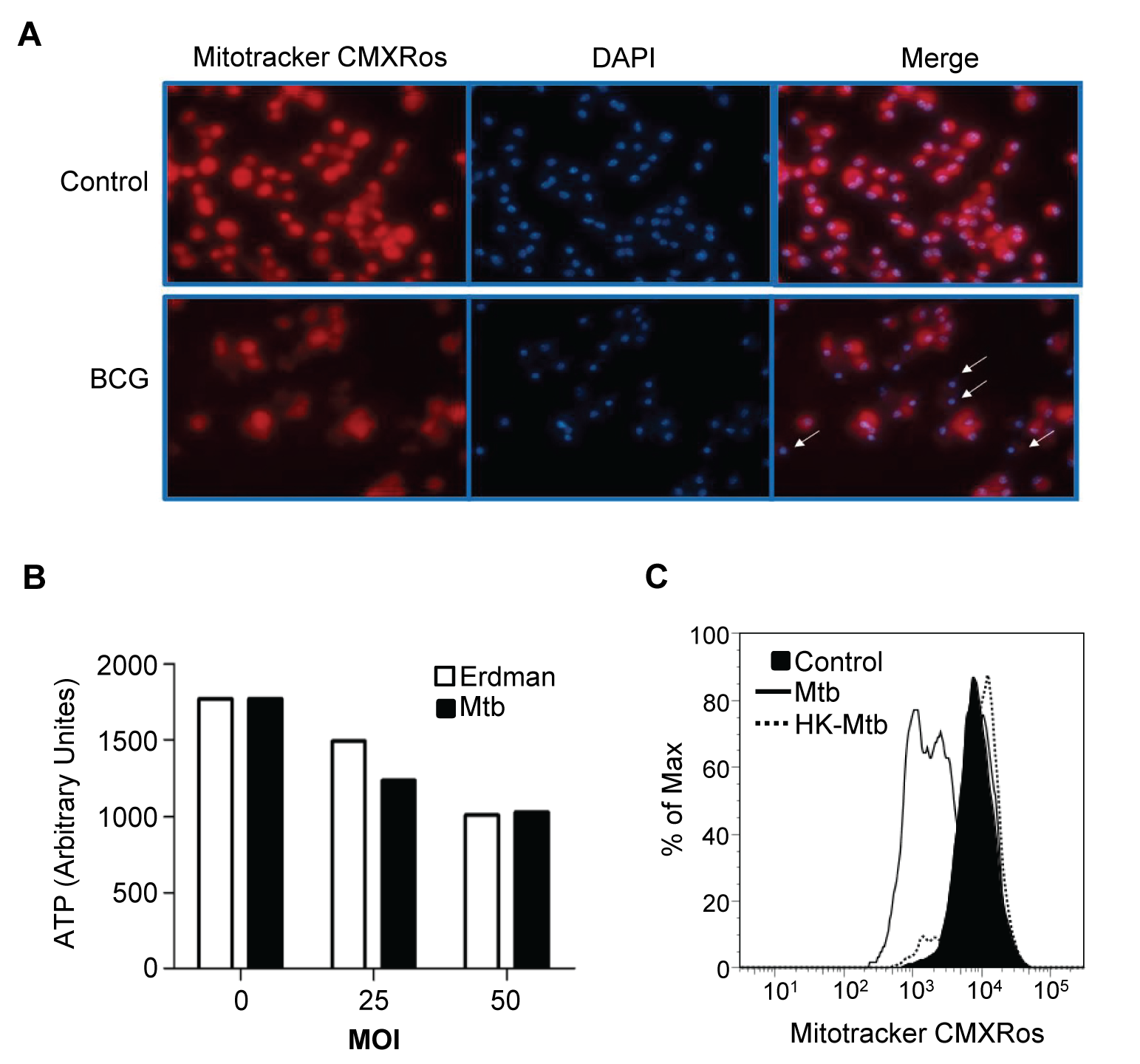

Supplement: Figure S4 — Mitochondrial injury in infected macrophages. (A) Macrophages were infected with BCG (3 h) and then stained with Mitotracker CMXRos and DAPI for examination by fluorescence microscopy. White arrows indicate cells with loss of Δψm demonstrated by relase of fluorescent dye from mitochondria (X, 400). (B) Macrophages plated in 24-well plates at 5×10^5^ per well were infected with BCG or Mtb Erdman for 3 h. Cells were washed with PBS twice and the cellular ATP level after was measured with ApoSENSOR™ ADP/ATP Ratio Assay kit (BioVision) following the manufacturer's protocol. (C) Macrophages were challenged with viable or heat-killed (HK) Mtb (3 h) and then probed with Mitotracker CMXRos for analysis by flow cytometry. (TIF) [file pone.0018367.s004.tif]

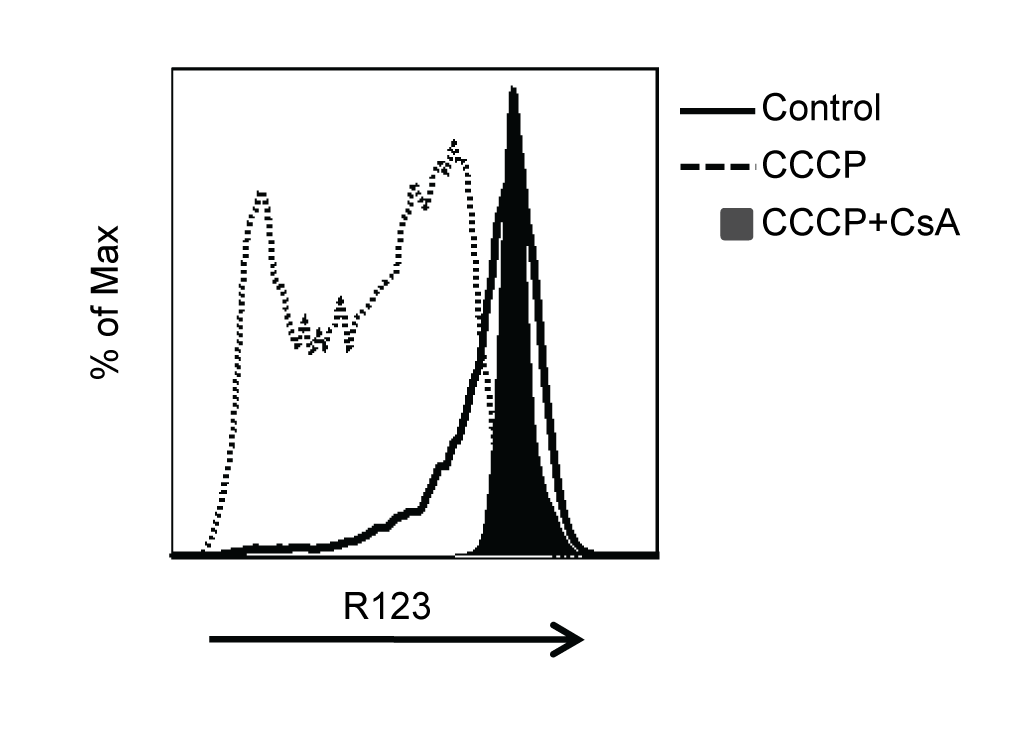

Supplement: Figure S5 — CsA blocks m -chlorophenylhydrazone-induced mitochondrial PT pore formation. Macrophages were treated with 20 uM of m-chlorophenylhydrazone (CCCP) in the presence or absence of of 5 uM CsA for 24 h and then stained with 10 uM of rhodamine 123 (R123) for 15 min. Cells were washed and the R123 fluorescence was assessed by flow cytometry. CCCP-induced mitochondrial PT pore formation as evidenced by low R123 retention by mitochondria was reversed by CsA treatment. (TIF) [file pone.0018367.s005.tif]

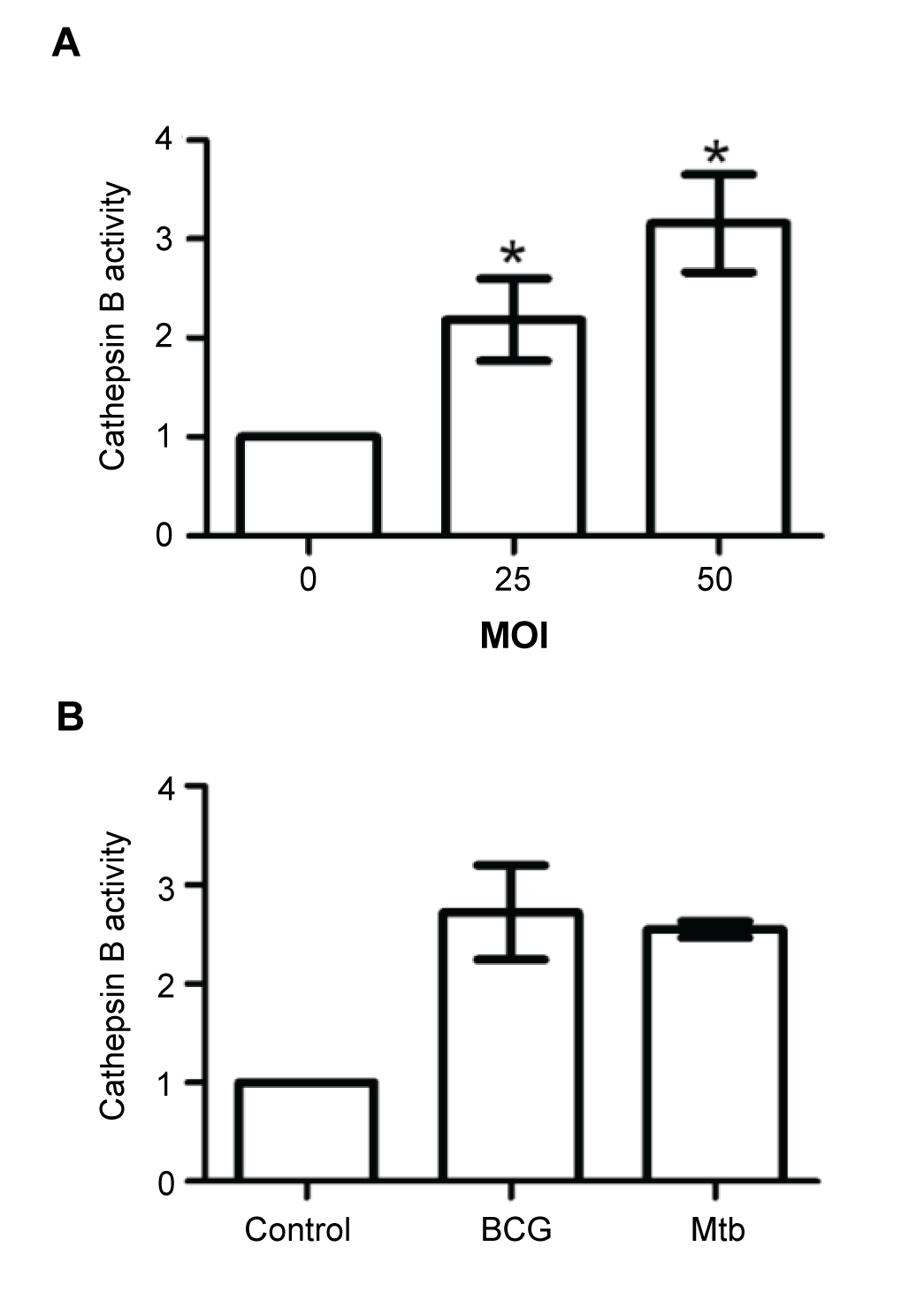

Supplement: Figure S6 — Induction of LMP by Mtb and BCG. (A) Macrophages were infected with Mtb (2 h) and then cytosolic extracts were prepared for measurement of cathepsin B activity indicative of LMP. Results are expressed as the fold increase of cathepsin B activity relative to uninfected (control) cells, normalized by the fold increase in LDH compared to uninfected cells (*P<0.05; error bars, ± SD). (B) Cytosolic cathepsin B activity in macrophages challenged with BCG or Mtb (*P<0.05). (TIF) [file pone.0018367.s006.tif]

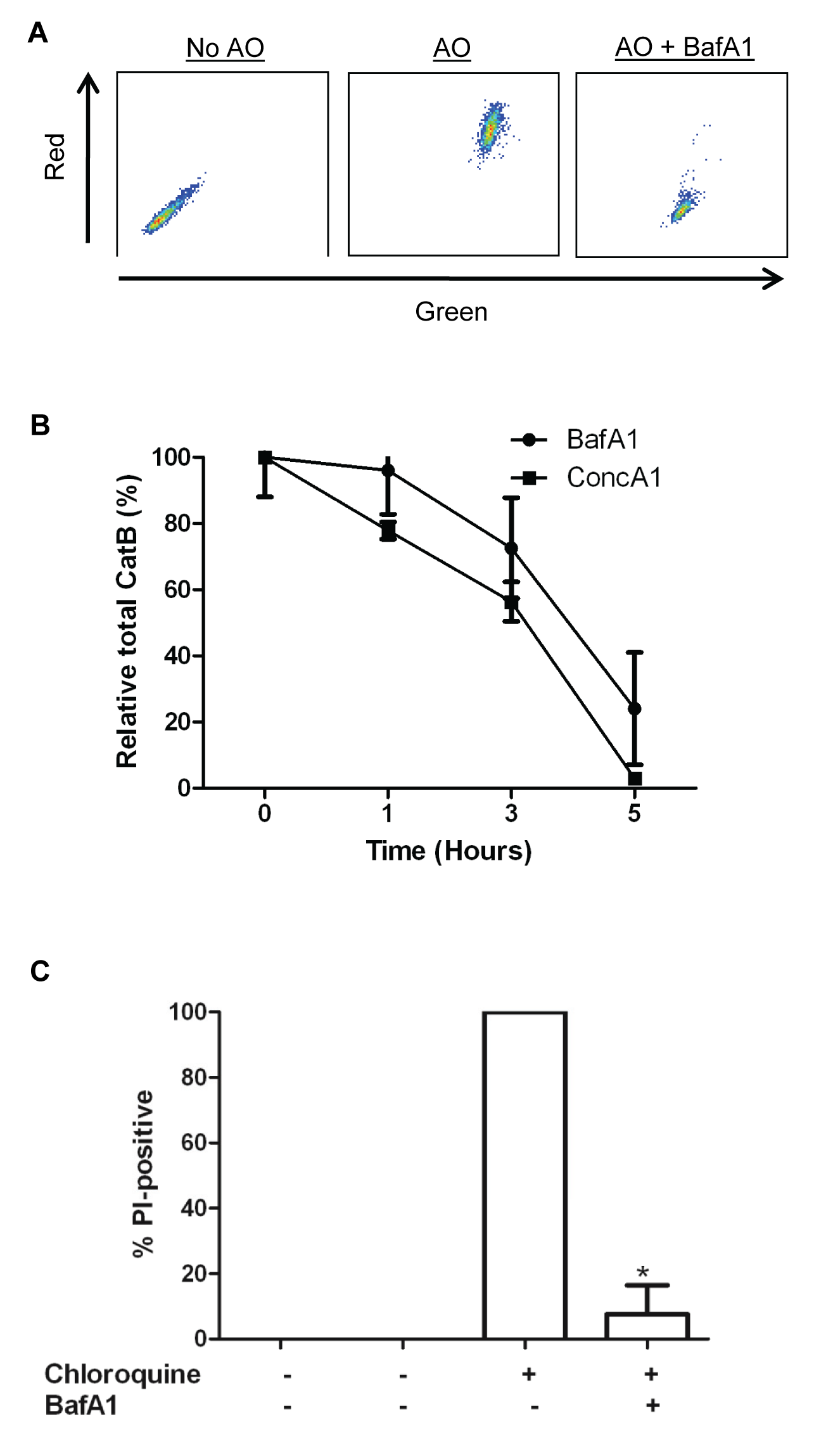

Supplement: Figure S7 — Bafilomycin A inhibits LMP and chloroquine-induced lysosomal cell death. (A) BafA1 abrogates acidification of lysosome leading to loss of acridine orange (AO). Cells were loaded with 5 µg/ml of AO in RPMI 1640 medium without serum for 15 min at 37°C. Cells were then treated with BafA1 for 3 h and red fluorescence (lysosomal AO) and green fluorescence (cytosolic AO) intensity was measured by flow cytometry. (B) BafA1 and concanamycin A (ConcA), both inhibitors for vacuolar-type ATPase, prevent cathepsin B activation. Macrophages were treated with 50 nM of BafA1 or ConcA for 1, 3, or 5 h, and total cellular cathepsin B activity was measured. Results are expressed as the mean cathepsin B activity ± SD. (C) BafA1 prevents chloroquine-induced lysosomal cell death. Macrophages treated as indicated with 50 nM of BafA1 and/or chloroquine at 100 µM overnight. Cell death was measured by PI-staining, expressed as the mean % PI-positive cells ± SD. (TIF) [file pone.0018367.s007.tif]

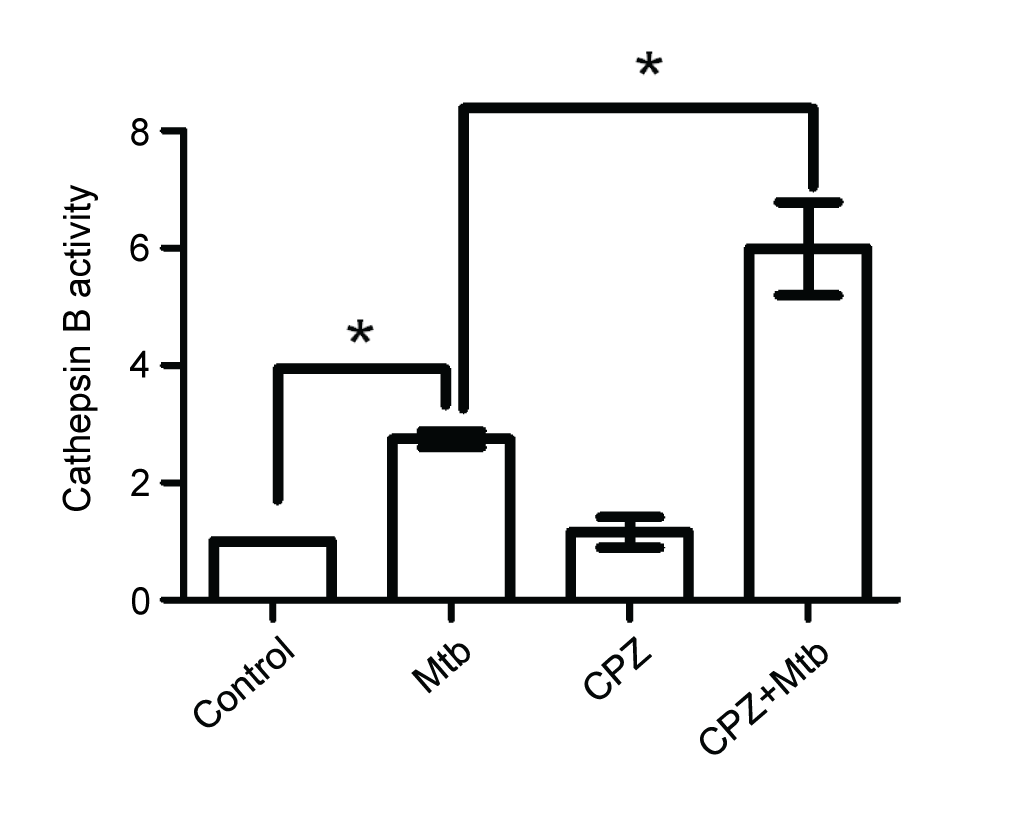

Supplement: Figure S8 — Chlorpromazine does not prevent LMP in Mtb-infected cells. Macrophages were pretreated with CPZ (10 µM) and challenged with with Mtb (2 h) in the presence of CPZ (10 µM). Cytosolic extracts were prepared for measurement of cathepsin B activity. Data are represented as mean fold increase of cytosolic cathepsin B (normalized to LDH as an indicator of cytosolic content) ± SEM (*p<0.05). (TIF) [file pone.0018367.s008.tif]

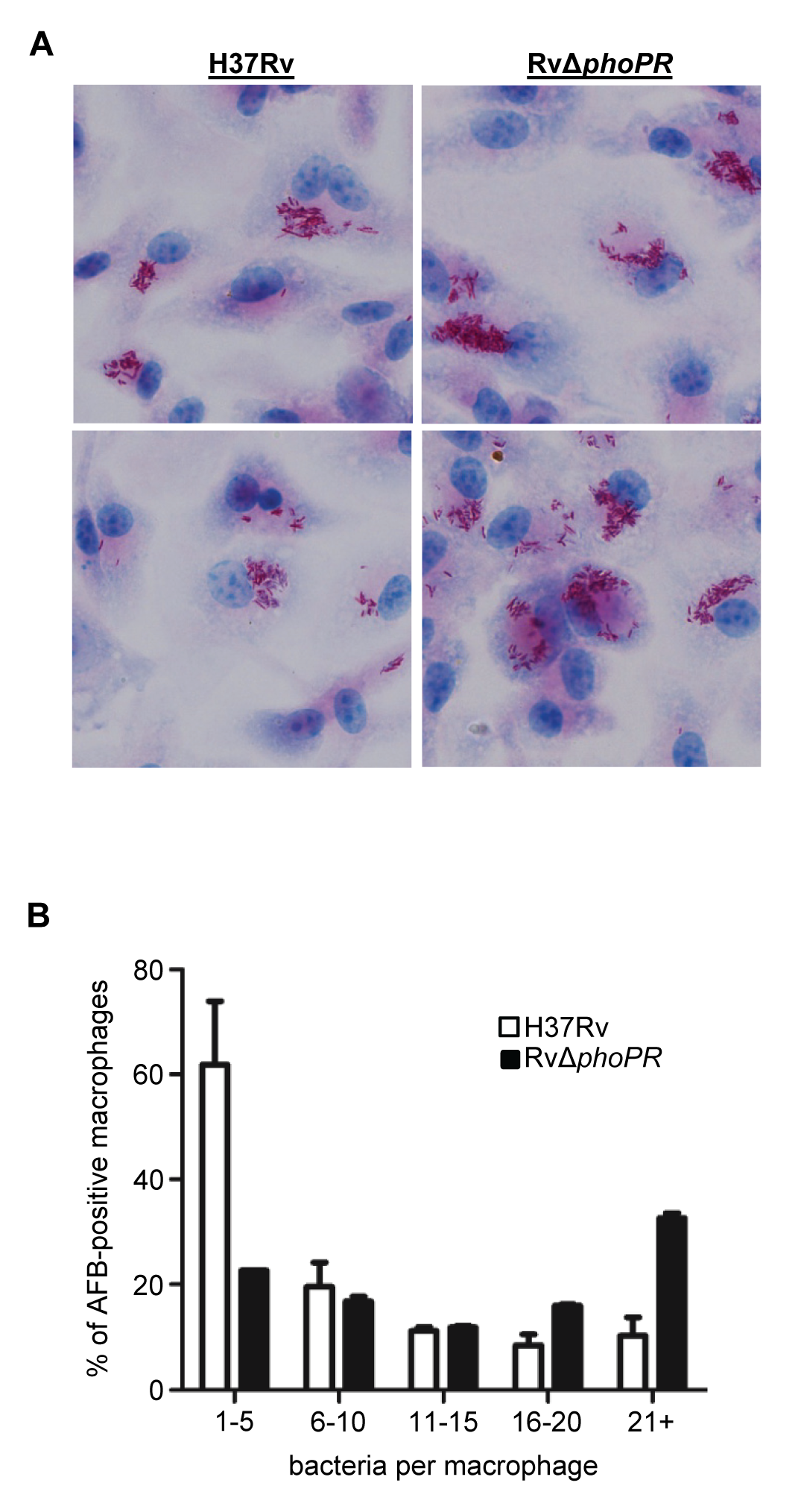

Supplement: Figure S9 — Macrophages engulf RvΔ phoPR more avidly than H37Rv. (A) Macrophages were challenged with H37Rv (right panels) or RvΔphoPR (left panels) at MOI 10. Unbound bacteria were removed by washing 3 h later and then slides were stained for acid fast bacilli. Magnification, X400. (B) The number of bacteria associated with macrophages was counted in 3 randomly selected fields in each of 2 Labtek chamberslide wells (∼300 macrophages per field) for macrophages challenged with H37Rv (open bars) or RvΔphoPR (filled bars). Results are displayed as the mean % macrophages infected with each designated range of bacterial number per chamberslide ± SD. (TIF) [file pone.0018367.s009.tif]

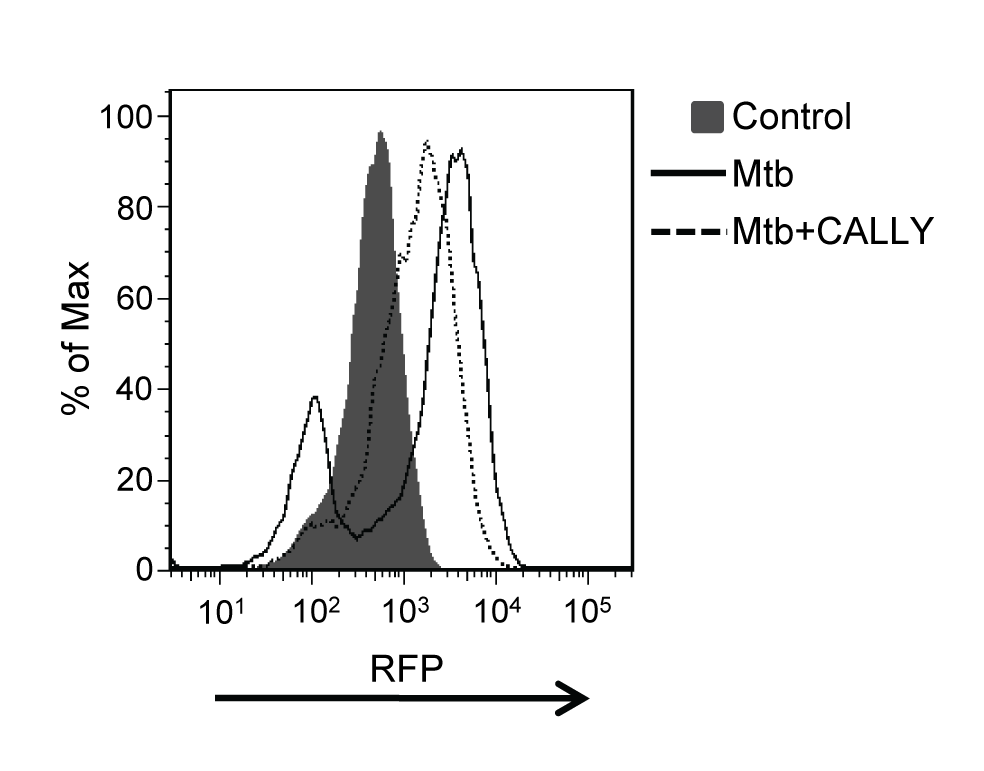

Supplement: Figure S10 — Cathepsin inhibitors reduce phagocytosis of Mtb by macrophages. Macrophages were pretreated with the cathepsin B inhibitor Ca-074-Me 40 µM plus the cathepsin L inhibitor Z-LLY-FMK 5 µM (CALLY) or control buffer and then challenged with Mtb H37Rv expressing red fluorescent protein (MOI 25, 3 hr). Phagocytosis of fluorescent bacteria was assesed by flow cytometry. (TIF) [file pone.0018367.s010.tif]
